# Prescribed fire is unlikely to reduce net PM_2.5_ emissions in most locations

**DOI:** 10.1073/pnas.2613722123

**Published:** 2026-06-29

**Authors:** Mark R. Kreider, Shawn P. Urbanski, Joseph Fargione

**Affiliations:** ^a^ Pacific Southwest Research Station, United States Department of Agriculture Forest Service, Redding, CA 96006; ^b^ Oak Ridge Institute for Science and Education, Oak Ridge, TN 37830; ^c^ Missoula Fire Sciences Lab, Rocky Mountain Research Station, United States Department of Agriculture Forest Service, Missoula, MT 59808; ^d^ The Nature Conservancy, Minneapolis, MN 55415

**Keywords:** emissions, prescribed fire, wildfire, air quality, PM_2.5_

## Abstract

Wildfire smoke poses a growing global health risk, largely from fine particulate matter (PM_2.5_) emissions. Prescribed fires, which are critical for maintaining resilient forests in many locations, can also reduce wildfire emissions in treated areas that later burn. However, prescribed fires also produce smoke, creating a tradeoff in their net impact on PM_2.5_ emissions. We develop a mathematical framework showing that, under most current conditions globally, prescribed fire emissions are rarely offset by reduced wildfire emissions and therefore increase PM_2.5_ emissions overall. An analysis of 73 prior study sites reveals that reported net emission reductions are often based on unrealistic assumptions, for example that all treatments are subsequently encountered by wildfire during their effective lifespan. Using empirically based estimates of expected encounter rates, prescribed fire increases net emissions at 99% of sites, with a median 10-year increase of 210% (IQR: 70 to 475%), and only 0.06 tons of wildfire emissions avoided per ton of prescribed fire emissions (IQR: 0.03 to 0.15). However, prescribed fires are intentionally conducted under favorable meteorological conditions, allowing smoke to disperse more safely than during wildfires. Thus, whether prescribed burning can reduce health risks while increasing emissions—and whether its emissions impact can be lowered beyond current practice—is a critical area for future study. Regardless, prescribed fires have multiple objectives and benefits; they remain essential for forest management and hazard reduction.

## Significance

Smoke exposure from wildfires increasingly poses serious global health risks. Prescribed fire is a critical component of treatments to reduce fuel loads and subsequent wildfire severity, but its net effect on emissions remains uncertain. We present a mathematical framework to determine under what conditions prescribed fire reduces total fine particulate (PM_2.5_) emissions. Our results show that, in most landscapes globally, prescribed fire increases emissions under current conditions. Additionally, nearly all studies reporting net emissions reductions rely on unrealistic assumptions that, when updated, reverse their findings. However, even if the emissions benefits of prescribed fires are sometimes overstated, prescribed fire has many other objectives and benefits and remains vital for forest and fire management.

Wildfire is a fundamental ecological process. However, its impacts are becoming more destructive and deadly in many locations globally, due to fire suppression, increased fuel loads, population growth in fire-prone areas, and climate change (1–4). Among the many consequences of this growing wildfire crisis, one of the most significant is the health impact from fine particulate (PM_2.5_) emissions (5–7). PM_2.5_ exposure from wildland fire is linked to increased mortality, worsened respiratory conditions, and increased rates of myocardial infarction (8–10), even at low concentrations (11, 12). These risks are projected to grow in the coming decades with intensified fire behavior from climate change, potentially causing millions of additional deaths from smoke exposure (5, 13, 14).

Prescribed fire is an important cultural and ecological process (15–17), and is a critical tool to reduce wildfire risk to forests and human communities (18–21). It has also been proposed as a way to reduce wildfire emissions (6, 22–24) and associated health impacts (25, 26) by lessening the intensity (22, 27) and potentially the spatial extent (28–31) of subsequent wildfires. However, prescribed fire also produces smoke (32, 33). Thus, evaluating the impact of prescribed fire on net emissions requires weighing these costs and benefits (22, 33, 34). Because the health impacts of PM_2.5_ emissions depend on factors including particle toxicology, how emissions are dispersed, and community responses, prescribed fire smoke may pose a lower health impact per unit of emissions than wildfire smoke if wildfire PM_2.5_ is more toxic (35, 36), prescribed fires are conducted under weather conditions that limit exposure to population centers (37, 38), and if the predictability of prescribed fire allows communities to better implement protective measures (39). Here, we focus on net emissions but note that the health impact per unit of emitted smoke remains an important and unresolved area for future research.

Studies on the net emissions impact of prescribed fire have mixed findings (22)—some report that prescribed fire reduces net emissions (23, 25–27, 38, 40–45), while others find increases in emissions or associated health risks (46–57). As a result, whether prescribed fires reduce overall emissions and health impacts—and under what conditions—remains an active area of research and debate (21, 22, 33, 34, 46, 58). Although prescribed fire has many objectives beyond emissions reduction, we focus on that outcome here; to clarify the current debate and evaluate how current and potential future prescribed fire practices can mitigate the impact of wildland fire emissions.

**Fig. 1.**
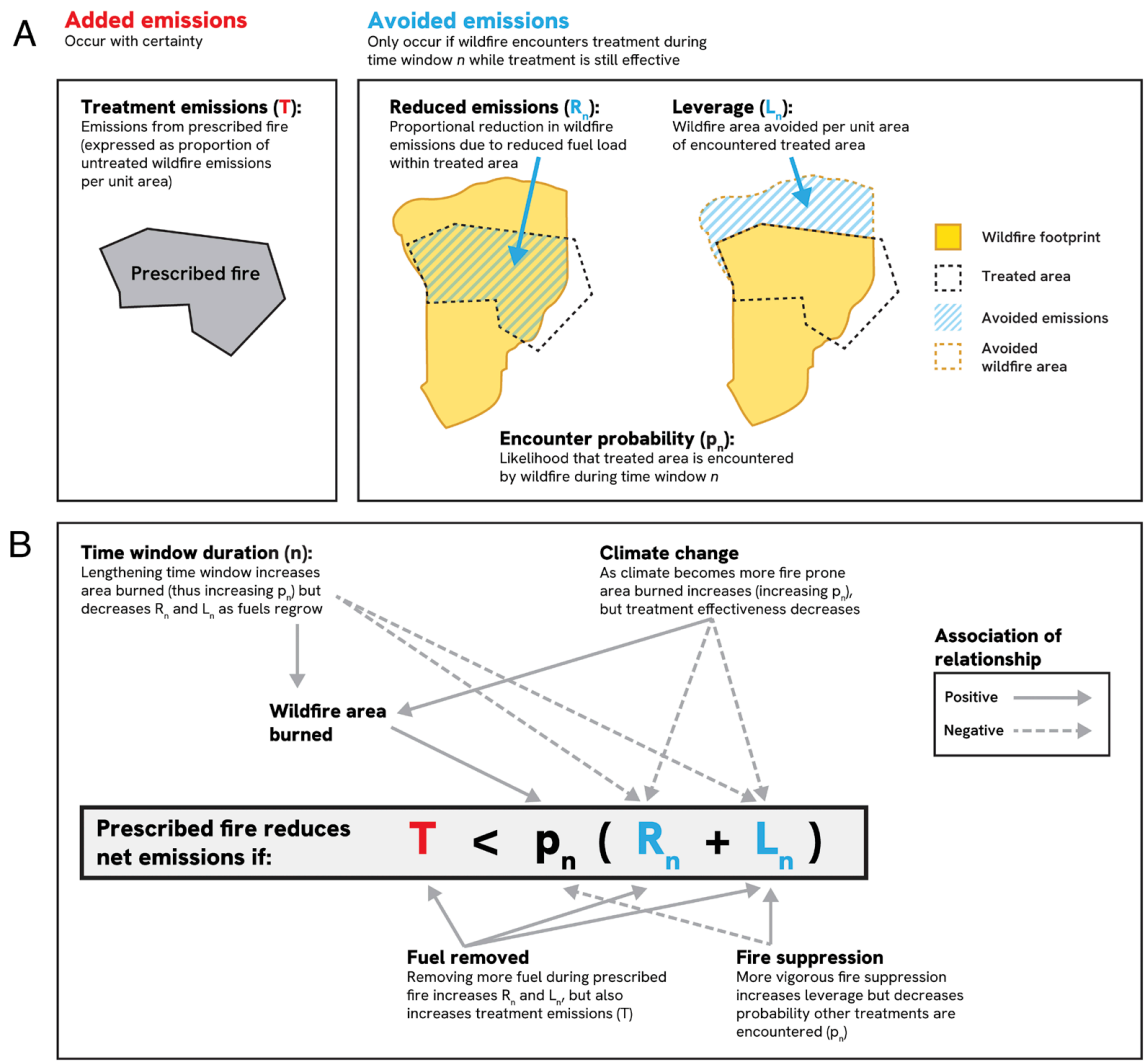
Conceptual figure of prescribed fires and impacts on net emissions, as a function of treatment emissions (T), encounter probability ($p_{n}$), emissions reduction proportion ($R_{n}$), and leverage ($L_{n}$). (*A*) Prescribed fire as both a cause of added emissions and potential avoided emissions. Note that potential avoided emissions only occur if a treatment is encountered by subsequent wildfire. (*B*) Prescribed fire reduces net emissions if $T<p_{n}\left(R_{n}+L_{n}\right)$ (Eq. **6**). External impacts on prescribed fire are shown, and how they influence whether net emissions are increased or decreased by prescribed fire. Note that parameters likely cannot be simultaneously maximized (e.g., climate change may increase $p_{n}$, but decrease the effectiveness of treatments).

Prescribed fire reduces net emissions only if it prevents more emissions in subsequent wildfires than it produces itself. Because prescribed burns are intentionally conducted to burn at lower intensity and with careful smoke management under favorable weather, they generally produce fewer emissions per area burned than wildfires (27, 45, 59). However, emissions from prescribed fire occur with certainty whenever an area is treated, while the emissions reduction benefits of prescribed fire occur only if a wildfire encounters the treated area during the period when the treated area has meaningfully reduced fuel loads (Fig. 1; 23, 26, 45, 48, 49)). Treatments are effective at mitigating fire behavior for a limited time, though the duration varies across ecosystems and is not well constrained. For example, in temperate forests of the western United States, prescribed fire treatments generally retain measurable effectiveness at reducing burn severity for 10 to 20 y (19, 60–62), though repeated prescribed fire can shift vegetation more permanently, for example moving closed canopy forests toward more open woodland structure (17, 63).

Emissions reduction benefits arise through two mechanisms: 1) reduced fuel loads, which lower wildfire intensity and emissions (19, 64), and 2) reduced wildfire extent, or “leverage”, which we define as the wildfire area avoided for each unit of treated area encountered by wildfire (28, 29, 46). Leverage can result from reduced fire spread or from easier control by firefighters, which may occur when treatments create safer locations for undertaking fire suppression and containment activities (31, 65). However, many treatments are designed primarily to reduce fire severity rather than area burned (19, 66), so leverage values near zero may be common (28, 46), especially in landscapes with low levels of treated area (31, 65). Even if the entire landscape were hypothetically treated, prescribed fire would reduce wildfire emissions per unit area burned, but would not prevent all subsequent wildfires (54, 65). Understanding the effect of prescribed fire on net emissions therefore requires accounting for both emissions from prescribed fire and subsequent wildfires.

We incorporate these fundamental mechanisms into a mathematical framework to quantify the change in total wildland fire emissions—those from both prescribed fire and wildfire—under the full range of conditions. While we largely focus on PM_2.5_ emissions, the framework is equally applicable to other pyrogenic emissions (e.g., carbon monoxide, volatile organic compounds). We extend this framework to the landscape scale (building on Kelp et al. 43), and incorporate leverage effects alongside emissions reductions (see Table 1 for variable summary). Note that all parameters described below vary spatially and temporally; however, they can be empirically measured or estimated for a given landscape and time window. The net change in emissions ($\Delta_{E}$) for a landscape is the proportional difference between emissions with prescribed fire ($E_{\mathrm{Rx}}$) and without prescribed fire ($E_{\mathrm{NoRx}}$):

[1] $$\Delta_{E}=\frac{E_{\mathrm{Rx}}-E_{\mathrm{NoRx}}}{E_{\mathrm{NoRx}}}.$$

**Table 1.** Summary of variables used in net emissions framework

Variable type	Variable	Description	Example units^*^	Equation
Measured	A	Area of landscape	ha	—
Measured	x	Per-unit-area emissions from prescribed fire treatment.	kg ha^−1^	—
Measured	y	Per-unit-area emissions from wildfire in untreated area.	kg ha^−1^	—
Measured	$z_{n}$	Per-unit-area emissions from wildfire in treated area (average value if a wildfire encounters treated area sometime within *n* years after treatment).	kg ha^−1^	—
Measured	Q	Proportion of landscape treated with prescribed fire	unitless	—
Chosen	n	Duration of time window considered	years	—
Calculated	$p_{n}$	Probability that a given treated point is encountered by wildfire within *n*-years. Assumed to be equivalent to the proportion of the landscape burned within *n-*years (i.e., the wildfire burn probability).	unitless	Calculated as treated area encountered by wildfire within *n*-years divided by total amount of treated area (67).
Calculated	$L_{n}$	Leverage (wildfire area prevented by treated area; average value if a wildfire encounters treated area within *n* years after treatment)	unitless [(prevented) ha per (encountered) ha]	Calculated as number of units of wildfire prevented for every unit of treated area encountered by wildfire (29)
Calculated	T	Treatment emissions; ratio of prescribed fire emissions to untreated wildfire emissions for a given location	unitless	$T=\frac{x}{y}$
Calculated	$R_{n}$	Average reduction in wildfire emissions when a wildfire burns an area that was treated within *n*-years.	unitless	$R_{n}=\frac{y-z_{n}}{y}$
Calculated	$E_{\mathrm{NoRx}}$	Landscape emissions in a scenario without prescribed fire (e.g., only wildfire emissions).	kg	$E_{\mathrm{NoRx}}={\mathrm{Ap}}_{n}y$
Calculated	$E_{\mathrm{Rx}}$	Landscape emissions in a scenario with prescribed fire (e.g., wildfire emissions and prescribed fire emissions).	kg	$E_{\mathrm{Rx}}=Q\cdot Ax+Q\cdot Ap_{n}y(1-R_{n}-L_{n})+(1-Q)(Ap_{n}y)$
Calculated; net emission result	Π	Ratio of avoided wildfire emissions to added prescribed fire emissions. E.g., for every ton of prescribed fire emissions, how many tons of wildfire emissions are avoided?	unitless	$\Pi =\frac{p_{n}\left(R_{n}+L_{n}\right)}{T}$
Calculated; net emission result	$\Delta_{E}$	Net emissions change because of prescribed fire. This value is a proportion: values < 0 indicate a reduction in emissions; values > 0 indicate an increase in emissions (e.g., 1.4 = 140% increase).	unitless	$\Delta_{E}=\frac{{E_{\mathrm{Rx}}-E}_{\mathrm{NoRx}}}{E_{\mathrm{NoRx}}}$ $=Q\left(\frac{T}{p_{n}}-\left(R_{n}+L_{n}\right)\right)$

^*^We provide example units for illustration purposes. Note that any area unit could be used in place of ha, any time unit could be used in place of year, and any emissions unit could be used in place of kg.

Without prescribed fire, expected emissions ($E_{\mathrm{NoRx}}$) for a landscape over a specified time period (n) are equal to the probability of wildfire occurrence over the time period ($p_{n}$) multiplied by the area of the landscape (A) multiplied by the average untreated wildfire emissions per unit area (y):

[2] $$E_{\mathrm{NoRx}}={\mathrm{Ap}}_{n}y.$$

With prescribed fire, expected emissions for a landscape can be broken into three components: emissions from the treatment itself, subsequent wildfire emissions from treated areas, and subsequent wildfire emissions from untreated areas. Emissions from prescribed fire treatments are equal to the product of the proportion of the landscape treated (Q), the landscape area (A), and the average prescribed fire emissions per unit area (x). On the treated portion of the landscape (Q), subsequent wildfire emissions equal the expected wildfire emissions (${\mathrm{Ap}}_{n}y$), reduced by two factors: 1) the proportional reduction in wildfire emissions within treated areas due to lower fuel loads ($R_{n}$; e.g., a value of 0.3 means treated-area wildfire emits 30% less than it would have without treatment), and 2) wildfire emissions avoided outside treated areas due to leverage ($L_{n}$; average units of wildfire avoided for each unit of prescribed fire encountered). With avoided wildfire due to leverage already accounted for, the remaining untreated portion of the landscape (1-Q) has emissions of $Ap_{n}y$. We assume that wildfire burn probability is equivalent to treatment encounter probability within the given time window [sensu (43, 46)]. Thus, the total emissions under the prescribed fire scenario are

[3] $$E_{\mathrm{Rx}}=Q\cdot Ax+Q\cdot Ap_{n}y(1-R_{n}-L_{n})+(1-Q)(Ap_{n}y).$$

We define the treatment emissions (T) as the ratio of prescribed fire emissions (x) to untreated wildfire emissions (y):

[4] $$T=\frac{x}{y}.$$

Evaluating Eq. **1** with Eqs. **2**, **3**, and **4** shows that the proportional change in net emissions for a landscape ($\Delta_{E}$) depends on just four varying parameters (T, $p_{n}$, $R_{n}$, and $L_{n}$) and one known, management-controlled parameter (Q):

[5] $$\Delta_{E}=Q\left(\frac{T}{p_{n}}-(R_{n}+L_{n})\right).$$

Regardless of the proportion of the landscape treated, prescribed fire only reduces net emissions ($\Delta_{E}<0$) if the added treatment emissions (T) are less than the avoided wildfire emissions, $p_{n}\left(R_{n}+L_{n}\right)$ (Fig. 1):

[6] $$T<p_{n}(R_{n}+L_{n}).$$

Additionally, the ratio (Π) of avoided wildfire emissions ($E_{\mathrm{avoided}}$) to added prescribed fire emissions ($E_{\mathrm{added}}$) is

[7] $$\Pi =\frac{E_{\mathrm{avoided}}}{E_{\mathrm{added}}}=\frac{p_{n}(R_{n}+L_{n})}{T}.$$

Note that the encounter rate of treatments ($p_{n}$) is driven primarily by wildfire extent; treating more area (i.e., increasing Q) expands both the treated area that wildfire intersects and the treated area it does not intersect, so the proportion encountered remains roughly constant. While encounter rate does not increase with area treated, leverage may be impacted by the proportion of the landscape treated (Q) as well as the spatial arrangement of treatments (65). These parameters interact to determine how prescribed fire may reduce the “effective” wildfire burn probability. This effective wildfire burn probability under the prescribed fire scenario ($p_{n}^{eff}$) can be calculated as the fraction of the landscape in the prescribed fire scenario burned by wildfire: the baseline amount of wildfire (${\mathrm{Ap}}_{n}$) minus the wildfire area avoided ($AQp_{n}L_{n}$), all divided by the landscape area (A). Thus, $p_{n}^{eff}=\frac{{\mathrm{Ap}}_{n}-AQp_{n}L_{n}}{A}$, or:

[8] $$p_{n}^{\mathrm{eff}}=p_{n}(1-QL_{n}).$$

Because it is not possible to exclude fire from more than 100% of the landscape, Eq. **8** implies inherent bounds on leverage values across a landscape: on average, each hectare of encountered treated area can prevent at most $\frac{1}{Q}$ hectares of wildfire. Like the ability of a treatment to lower emissions in a subsequent wildfire ($R_{n}$), the strength of leverage also decreases over time (68).

We derive the mathematical framework from probabilistic encounter rates over a short timeframe (n), though the same form also emerges from long-term sequences of repeated wildfires and prescribed fires (*SI Appendix*, *Supplemental Methods*). Because the health burden of PM_2.5_ depends on smoke dispersion and population exposure, we extend the framework to compare the per-unit health impacts of wildfire and prescribed fire emissions (*SI Appendix*, *Supplemental Methods*). This yields a threshold for the relative impact required for prescribed fire to reduce overall health burdens, which is identical to Eq. **7**: if prescribed fire adds more emissions than it avoids, the per-kilogram health impact must be lower by exactly this ratio for prescribed fire not to increase health impacts (see *SI Appendix*, *Supplemental Methods* for derivation).

Using this framework, we examine how expected net PM_2.5_ emissions vary across global conditions. Specifically, we address three questions: 1) Under what conditions does prescribed fire reduce net emissions? 2) What are the empirical ranges of parameter values (T: treatment emissions; $p_{n}$: encounter probability; $R_{n}$: proportional reduction in wildfire emissions from reduced fuel loads; and $L_{n}$: leverage) across landscapes globally? 3) Can this framework explain the mixed findings on whether prescribed fire reduces net emissions? While we focus on PM_2.5_ emissions from wildland fires (both prescribed fires and wildfires), this framework is applicable to other emissions produced by fire (e.g., CO_2_). Accordingly, for Question 3, we consider studies reporting the net effects of prescribed fire on any pyrogenic emission, not solely PM_2.5_. However, it would be inappropriate to draw conclusions about net CO_2_ flux from our study, as severe wildfire can suppress future sequestration for decades (69, 70). Treatments that reduce fire severity would allow continued sequestration and may yield net CO_2_ reduction benefits (71)—even if, as our results suggest, prescribed fire increases net CO_2_ emissions from combustion. Our results thus apply to pyrogenic emissions only; carbon fluxes such as postfire sequestration or decomposition are beyond the scope of this analysis.

## Results

### Net Emissions Reductions Are Only Possible Under Rare Conditions.

**Fig. 2.**
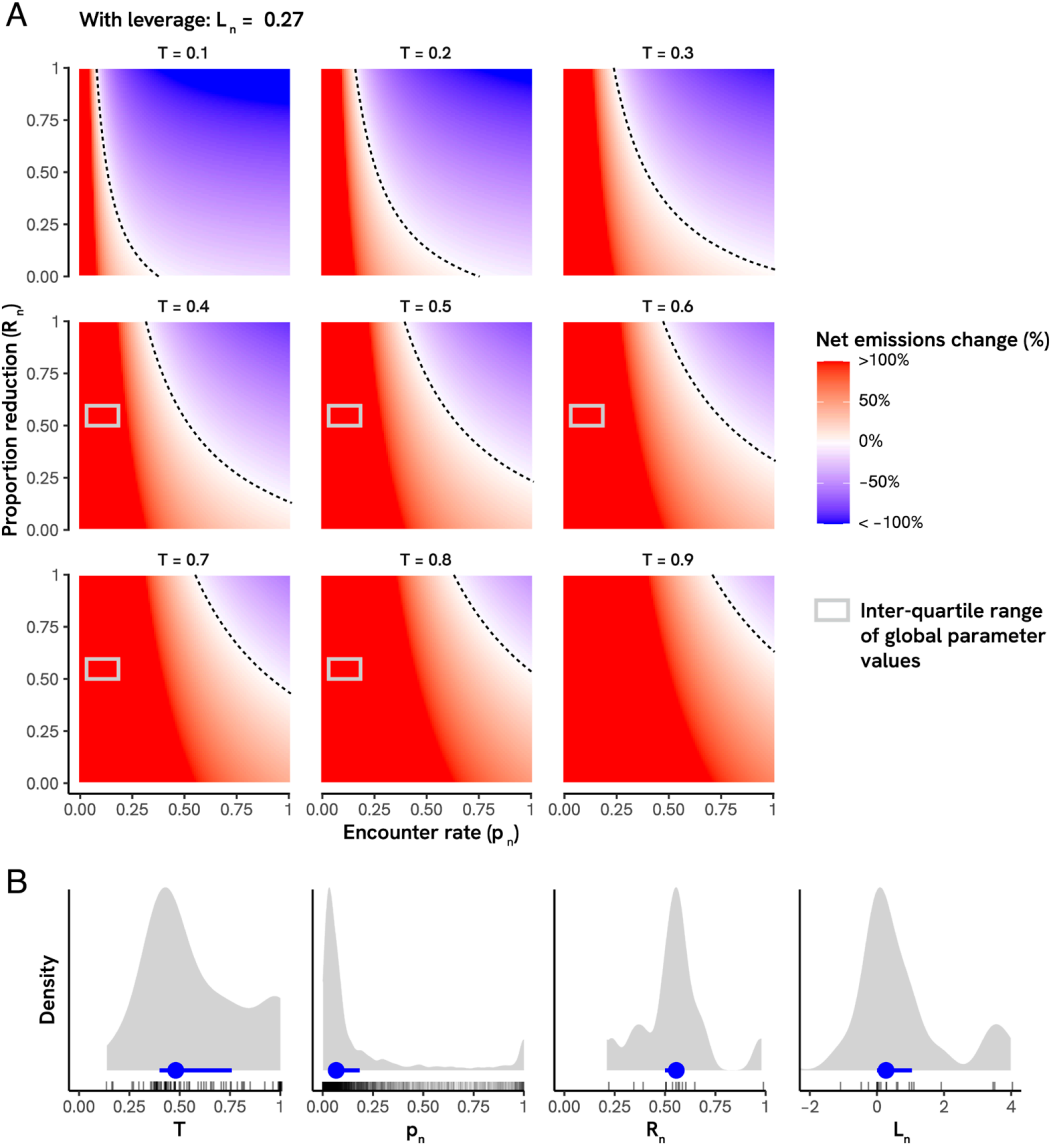
Net emissions change across a range of parameters ($p_{n}$, $R_{n}$, $L_{n}$, T). (*A*) Net emissions change across possible ranges of $p_{n}$, $R_{n}$, and T. The dashed line depicts the point at which net emissions change is 0. Gray rectangles show the parameter space which falls within the interquantile range (25 to 75%) from empirically estimated values of (n = 10 y) $p_{n}$, $R_{n}$, and T. $L_{n}$ is set to its estimated median value from empirical studies ($L_{n}$ = 0.27). Net emissions change greater than 1 (i.e., >100%) are all depicted as the same shade of red. (*B*) Global empirical distributions of each parameter, with median and interquartile range (25 to 75%) shown in blue point or line, respectively. Due to smoothing of kernel density distributions, the interquartile ranges may appear to cover less than 50% of the distribution density; tick marks show the actual location of empirical data observations.

Our framework shows that, under most conditions, prescribed fires increase net emissions (Fig. 2*A* and *SI Appendix*, Fig. S1). For example, if leverage is low and treatment emissions and reductions from treatment are moderate (T = 0.5; $R_{n}$ = 0.5), prescribed fire will increase net emissions unless the expected encounter probability of treatments ($p_{n}$) is >50% (Fig. 2*A* central panel). Conversely, at low encounter probabilities, net emissions reductions become highly unlikely, even if all other parameters are optimal (*SI Appendix*, Fig. S1). Even under optimistic assumptions where treatments only produce 30% the emissions of wildfires (T = 0.3), wildfire emissions are reduced by 70% in treated areas ($R_{n}$ = 0.7), and 3 ha of wildfire are avoided for each hectare of treated area encountered by wildfire ($L_{n}$ = 3), prescribed fires would still increase net emissions if encounter probabilities are not over ~8%. For comparison, median 10-y encounter probabilities in the United States and globally are currently ~7% (67) (*SI Appendix*, Fig. S2). This framework implies that net emissions reductions from prescribed fire are possible only in select locations with high encounter probabilities, low treatment emissions, and high treatment effectiveness ($R_{n}$ and $L_{n}$).

### In Most Locations Globally, Current Conditions Do Not Support Net Emissions Reductions.

Parameter values (T, $p_{n}$, R, $L_{n}$) vary greatly across vegetation types and through time, and empirical data to estimate them are sparse in many ecosystems. However, existing data on global empirical ranges of $p_{n}$, T, $R_{n}$, and $L_{n}$ suggest that only a small fraction of locations support net emissions reductions from prescribed fire on average (Fig. 2*B* and *SI Appendix*, Fig. S3).

Of all parameters, encounter probability ($p_{n}$) is the best quantified. We use 10-y encounter probabilities, as treatments are typically effective over this timeframe (19) (*Discussion*). In the contiguous U.S., 10-y encounter probabilities across National Forests are low (median: 5%; IQR: 1 to 14%; full range: 0 to 43%) (Fig. 2*B*) (67). Globally, we estimated 10-y encounter probabilities using monthly area burned rasters (72), and extracted values for the locations of global prescribed fire records (73) (*SI Appendix*, Fig. S2). The median 10-y reburn probability of these locations is 7% (IQR: 3 to 18%) (*SI Appendix*, Fig. S2), though some regions such as occur in Brazil, South Africa, or Russia may exceed encounter probabilities of 50%.

Treatment emissions (T) are less well quantified, with no datasets allowing worldwide estimates. To create an estimate of treatment emissions variability, we used several sources. First, we used reported treatment emissions from 24 sites globally (*SI Appendix*, Table S1), with a median T = 0.39 (IQR: 0.38 to 0.47). In the United States, EPA state-level emissions inventory data (74) indicate a median of T = 0.67 (IQR: 0.43 to 0.93) (*SI Appendix*, Fig. S4). Finally, we used two other studies which estimated T values of 0.65 (56) and 0.47 (59). Together, treatment emissions are a median of 48% of wildfire emissions in the same location (T = 0.48; IQR: 0.40 to 0.76).

To estimate the proportional reduction in wildfire emissions in treated areas ($R_{n}$), we used values from studies estimating net emissions change (*SI Appendix*, Table S1), excluding those reporting $R_{n}$ = 0 or $R_{n}$ = 1. This yielded five studies with a median value of $R_{n}$ = 0.54 (IQR: 0.50 to 0.58). A meta-analysis of carbon emissions from treated and untreated forest stands in the western United States (64) found a median $R_{n}$ = 0.56 (IQR: 0.48 to 0.59), and one additional study (56) reported a value of $R_{n}$ = 0.70. Together, wildfire emissions in treated areas are a median of 56% lower than untreated wildfire emissions ($R_{n}$ = 0.56; IQR: 0.50 to 0.60). These estimates are aggregated across varying encounter windows rather than a standardized time period; $R_{n}$ presumably decreases with time since treatment (19).

Leverage ($L_{n}$) is difficult to quantify because it is a counterfactual: how much wildfire would have burned had a location not been treated? In fire modeling of 14 large U.S. wildfires (29), every hectare of encountered treated area avoided a median of 0.28 ha of wildfire area ($L_{n}$ = 0.28, IQR: 0.04 to 1.02). In a global analysis of leverage from previous wildland fire (28), half of sites had no leverage ($L_{n}$ = 0), including sites in Canada, Spain, and the western U.S. Sites in Australia and Portugal had higher leverage (1.9 and 3.5 respectively), however, these estimates include the impact of both prior prescribed fire and wildfire, likely overestimating the leverage from prescribed fire alone. Another analysis from Portugal found that leverage from prescribed fires alone was not statistically different from zero (30). Like $R_{n}$ values, reported $L_{n}$ values have inconsistent time windows, ranging from 1 to 8 y following treatment (28–30); leverage presumably decreases over time as fuels regrow (19). Considering all available studies, global estimates show a median leverage of $L_{n}$ = 0.27 (IQR: 0.00 to 1.04). While locations with lower fuel (e.g., mediterranean or savanna systems) may have moderate leverage, higher-fuel locations (e.g., temperate or boreal forest) may have nonexistent or, at best, modest leverage (46).

At global median values of parameters ($p_{n}$ = 0.07, T = 0.48, $R_{n}$ = 0.56, $L_{n}$ = 0.27), prescribed fires increase PM_2.5_ emissions by 634% over a 10-y period (Eq. **5**). Similarly, only 0.115 tons of wildfire emissions are avoided for each ton of prescribed fire emissions (Eq. **7**), implying that for prescribed fire to yield a health benefit, the health impacts per kg of prescribed fire smoke would have to be 8.7 times lower than that of wildfire smoke (i.e., $\frac{1}{0.115}$; *SI Appendix*, *Supplemental Methods* and Fig. S5). Evaluated across global 10-y burn probabilities and with other parameters at their median, prescribed fire yields net emissions reductions only where 10-y encounter probabilities exceed 58% (*SI Appendix*, Fig. S3). Even if other parameters are set at their 95% favorable percentile values (T = 0.27, $R_{n}$ = 0.80, $L_{n}$ = 3.51), a 10-y encounter probability of at least 6.2% would be required for prescribed fire treatments to reduce net PM_2.5_ emissions—a value higher than the median 10-y encounter probability across national forests in the western United States (67) (*SI Appendix*, Fig. S3).

### Most Studies Finding Net Emissions Reductions Do Not Account for Encounter Probability.

**Fig. 3.**
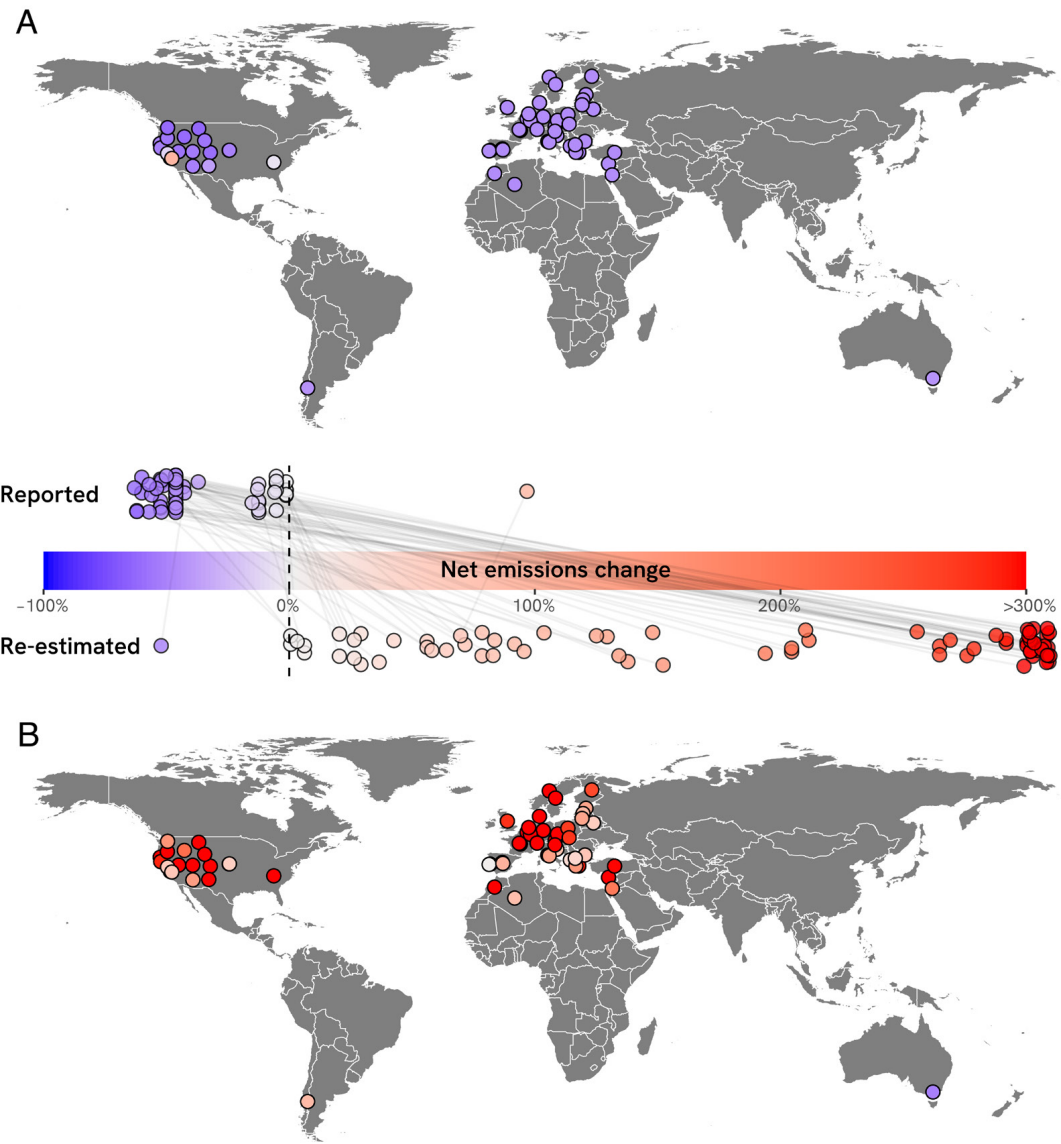
Net emissions change of global empirical studies. (*A*) Reported net emissions change of global studies. (*B*) Re-estimated net emissions of studies when parameters are updated to empirically supported values. See *SI Appendix*, Table S1 for complete reported and re-estimated parameter values of studies. Geographic points are jittered for studies with multiple estimates for the same location. Some studies (26, 27, 44, 45, 47–52, 56, 57) were not included in the analysis because parameters could not be extracted or inferred from the published data.

We analyzed studies that reported changes in net pyrogenic emissions (carbon or PM_2.5_) as a result of prescribed fires, where the four parameters ($p_{n}$, T, $R_{n}$, and $L_{n}$) could be derived from the reported data. We evaluated four studies (40, 41, 53, 75) from Hunter and Robles’ review on prescribed fire effects (22) as well as seven other recent studies (23, 25, 38, 42, 43, 76, 77). In total, these 11 studies report net emissions change for 73 locations, including the United States, Argentina, Australia, Europe, North Africa, and the Middle East (Fig. 3).

A single study (53) found that prescribed fire increased net emissions, largely because the majority of treatments were not encountered by wildfire. The remaining 72 study locations reported net emission reductions from prescribed fire (Fig. 3 and *SI Appendix*, Table S1). However, nearly every study reporting net reductions assumed that treated areas would be encountered by wildfire with certainty ($p_{n}$ = 1; *SI Appendix*, Table S1). Several also assumed high leverage values beyond empirically documented ranges (e.g., $L_{n}$ ~ 5 in refs. 25 and 41), omitted treatment emissions (T = 0 in ref. 23), or assumed that prescribed fire eliminated all subsequent wildfire emissions in treated areas ($R_{n}$ = 1 in refs. 38 and 40) (*SI Appendix*, Table S1).

We re-estimate net emissions change for all studies, using empirical values for 10-y encounter probability (67, 72) for all studies reporting encounter probabilities of 1, and revised T, $R_{n}$, and $L_{n}$ values where studies made unrealistic assumptions as described above or did not include the effect of $R_{n}$ or $L_{n}$. *SI Appendix*, Table S1 lists updated parameter values. With these updated values, 99% of study locations (72 out of 73) show net emissions increases from prescribed fire (Fig. 3 and *SI Appendix*, Table S1). Overall, sites have a median net emissions increase of 210% (in contrast to a median net emissions decrease of 46% as reported in the studies) with only 0.062 tons of wildfire emissions avoided for each ton of prescribed fire emissions. Thus, for prescribed fire to yield a health benefit, the health impacts per kg of prescribed fire smoke would have to be 16.1 times lower (IQR: 6.8 to 39.8) than that of wildfire smoke (i.e., $\frac{1}{0.062}$; *SI Appendix*, *Supplemental Methods*).

Of the locations initially reporting net decreases, only one location had a re-estimated decrease in net emissions—a site in dry Eucalyptus forest in south-eastern Australia with a much higher encounter probability ($p_{n}$ = 0.5) than other sites (76). Even if all study sites experienced a hypothetical doubling in 10-y encounter probability [i.e., an extreme increase in wildfire extent because of climate change (78)], 90% of sites (66 out of 73) would still yield net emissions increases.

## Discussion

Prescribed fire reduces net emissions only when treatment emissions are lower than the emissions avoided in subsequent wildfire (Fig. 1*B*). Yet, under current levels of wildfire, most treatments are never encountered by wildfire during their effective lifespan (67, 79) and therefore provide no emission reduction [though they provide many other benefits (22)]. Despite this evidence, many studies on net emissions change still assume that all treatments will be “used” in a wildfire (e.g., refs. 26, 44) (*SI Appendix*, Table S1). This assumption systematically biases studies toward finding net emissions reductions (54, 55) (Fig. 3): every study assuming a 100% encounter probability reported net decreases (*SI Appendix*, Table S1). For example, Wiedinmyer and Hurteau (40) concluded that prescribed fire could lower forest carbon emissions from fire across the western United States by between 37% and 63% (*SI Appendix*, Table S1). However, as noted by a subsequent comment (55), they assumed that treatments would always be encountered by wildfire ($p_{n}$ = 1) and would fully eliminate subsequent wildfire emissions ($R_{n}$ = 1). When these assumptions are replaced with realistic values, the result is reversed—prescribed fire increases, rather than reduces, overall emissions within their study area. It is important to note that increasing the area treated with prescribed fire does not increase encounter probability; doubling the area treated doubles both the areas that will and will not be encountered by wildfire, leaving the probability of encounter unchanged. Encounter probability can only be increased by increasing wildfire occurrence, either in absolute terms, or by locating treatments in areas with higher wildfire probabilities.

Several studies finding net emissions reductions use leverage values well above empirical ranges. For example, Burke et al. (25) assume in one scenario that every ha of prescribed fire prevents 4.9 ha of wildfire, citing ref. 29. However, 4.9 is not a leverage estimate but the ratio of wildfire area prevented (1.37 ha) to wildfire area promoted (0.28 ha) by one ha of prescribed fire (1.37/0.28 = 4.9; “promoted” fire reflects situations where treatments increase spread rates, for example by increasing grass cover). The actual leverage reported in ref. 29 is 1.1 (1.37 minus 0.28 = 1.1), and this reflects only the most successful fires; the median across all fires is just 0.28. Correcting this value greatly reduces—or reverses—their reported net emissions reductions. Finally, several studies reporting emissions or health benefits from prescribed fire (23, 80) omit emissions from prescribed fire itself (T = 0), which guarantees an apparent emissions reduction whenever $R_{n}$ and $L_{n}$ are positive, and therefore biases results. Assessing the emissions benefits of prescribed fire requires consideration of both the reductions in future wildfire emissions as well as added emissions from treatments.

Our findings are consistent with other studies that previously showed that prescribed fire likely increases pyrogenic emissions in most locations (46–57). While others have raised similar concerns, our framework distills studies to their underlying parameter assumptions, enabling direct comparison and revealing how frequently encounter probability of treatments is not accounted for. Not accounting for encounter likelihood risks misleading decision-makers and contributes to confusion in the literature. Our analysis suggests that much of the inconsistency in reported net emissions outcomes arises from studies that ignore encounter probability, and, to a lesser extent, assume unrealistic leverage values. When empirically grounded values are applied, nearly all studies show net emissions increases.

While 99% of sites showed net emissions increases under empirically derived parameter values (Fig. 3), one site still had net decreases—a highly fire-prone location in Australia (76) with 10-y encounter rates of ~50%. This pattern aligns with other studies not included in our analysis because we could not derive parameters from their presented data (27, 45), which also found net emissions reductions from prescribed fires even after accounting for encounter probability. These studies occurred in savannas in Brazil and Australia, areas characterized by both high fire frequency (i.e., high encounter rates) and strong leverage effects (45). Our results suggest that landscapes with exceptionally high encounter rates or leverage are the only settings where prescribed fire can be expected to consistently reduce net emissions from wildland fire (*SI Appendix*, Fig. S3). Even if climate change nearly doubles area burned in the future (78), the resulting increase in encounter probability may still be too low to reduce net emissions in most locations and conditions (*SI Appendix*, Figs. S6 and S7).

Net emissions reductions are theoretically possible in locations with modest encounter probabilities (<15%), such as much of the western United States, if all other parameters are simultaneously highly favorable (*SI Appendix*, Fig. S6). In practice, however, inherent tradeoffs make such combinations unlikely (Fig. 1*B*). For example, treatment effectiveness ($R_{n}$ and $L_{n}$) increases with greater fuel removal (66, 81), but removing more fuel requires higher treatment emissions (T), offsetting benefits. Climate change may increase wildfire extent (1), and thus encounter probabilities, however, more fire-prone conditions can reduce treatment effectiveness (82) (but see refs. 19, 83), lower leverage (84), and narrow safe windows for prescribed burning (85). Stronger fire suppression can increase leverage but would simultaneously reduce wildfire extent and encounter probability. Extending the time window for encounter probability (e.g., changing n from 10 to 20 y) can increase $p_{n}$ (67), but treatments lose effectiveness at longer windows (reduced $R_{n}$ and $L_{n}$) (19). Additionally, it is possible that at higher fractions of the landscape treated (larger Q), emergent effects could increase average leverage (86). However, because leverage is defined as conditional on being encountered by wildfire, it does not inherently rise simply because more area is treated. Moreover, Eq. **8** shows that average leverage cannot exceed $\frac{1}{Q}$ [i.e., at best, treatments can substitute prescribed fire for wildfire, though this theoretical limit has never been observed in forested ecosystems (46)], underscoring the inherent limits of potential wildfire emissions reductions even with substantially higher treated area. Together, these tradeoffs suggest that net emissions reductions from prescribed fire may be unlikely in many locations, regardless of attempts to optimize individual parameters.

While we derive and illustrate the framework over relatively short timescales, the same mathematical form emerges when extended to longer windows (e.g., >100 y) with repeated prescribed fire (*SI Appendix*, *Supplemental Methods*). In a scenario with repeated treatments, forest structure would likely become increasingly fire-resilient (87, 88), suggesting that treatment emissions (T) might decline and treatment effectiveness ($R_{n}$ and $L_{n}$) might rise over time. Process-based forest simulation models could help assess whether such shifts might eventually produce net emissions reductions, and over what timescales and in which ecosystems. However, one such recent paper using forest simulation modeling over a 100-y period in California found that prescribed fire increased net emissions even over this longer timeframe (57). Over long time periods, repeated prescribed fire may create landscapes akin to those in precolonial North America, where Indigenous-managed, low-intensity fires maintained open, resilient conditions (16, 20). However, one study (89) estimates that annual PM_2.5_ emissions in California prior to 1800 were 1,281 Gg, more than six times higher than the 2000–2023 average of 206 Gg (90). These pre-1800 annual emissions exceed even the major wildfire years of 2020 and 2021, which emitted 1,071 and 975 Gg of PM_2.5_, respectively (91). Thus, while prescribed fire clearly helps to restore resilient forest structure and mitigate the likelihood of high-severity fire (20, 69), it may not also reduce net emissions. Our framework shows how, even over long timescales, most prescribed fire treatments will not be “used” in a wildfire during their effective lifespan, so the fundamental trade-offs between prescribed fire and wildfire emissions persist.

Although we focus on prescribed fire, our framework applies to other forest treatments. Thinning can remove fuel and increase treatment effectiveness ($R_{n}$ and $L_{n}$) (19) with little or no treatment emissions (T), increasing the chance of a net reduction. However, follow-up burning of fine fuels left after thinning leads to the most effective treatments (19, 66, 81, 83, 88), suggesting that “thin plus burn” treatments may offer the best path to net emissions reductions by lowering T while maintaining high $R_{n}$ and $L_{n}$. For example, in a recent simulated analysis of the impacts of forest management on air quality (24), thinning accounted for the majority of the reduction in health risk, while increasing levels of prescribed fire appeared to erode those gains.

Our equations also apply to other pyrogenic emissions (e.g., CO, NO_x_, VOCs) (90), provided parameters T and $R_{n}$ can be calculated. While the framework can assess net CO_2_ emissions from combustion, it does not include postfire carbon fluxes. Because high severity wildfire can suppress carbon sequestration for years, treatments that reduce subsequent wildfire severity may have net carbon benefits through higher postfire sequestration (69, 92; but see 88), even if pyrogenic carbon emissions increase.

Our framework can also help guide future research on prescribed fire emissions. We encourage researchers to report parameter values and model assumptions (22), which are often difficult to infer from publications. Quantifying spatial variability in parameters remains a critical gap: encounter probability is reasonably well quantified (67, 72, 79), but how other parameters vary across landscapes and biomes is less well understood and is critical for identifying where prescribed fire is most likely to reduce net emissions. Treatment effectiveness ($R_{n}$ and $L_{n}$), for example, may depend strongly on treatment extent (Q) and spatial configuration (65, 86). Improved spatial quantification could also reveal how parameters covary and whether they can be optimized simultaneously: for instance, whether certain ecosystems, such as fire-prone savannas, naturally allow for greater potential emissions reductions.

Temporal dynamics of emissions reduction potential also warrant greater attention. Treatment effectiveness ($R_{n}$ and $L_{n}$) are time-dependent, yet little is known about how they change through time. Although fuel treatments can reduce fire severity for multiple decades (19), PM_2.5_-reduction benefits may follow a different timeline: duff and coarse woody debris, which produce more PM_2.5_ per unit mass (93), reaccumulate far more slowly than fine fuels (88). Because prescribed fire provides multiple socioecological benefits (19, 20, 22, 81, 94) that do not necessarily covary through time, treatment effectiveness cannot be captured with a single duration. Rather, we encourage research that evaluates specific benefits and how each one’s effectiveness changes over time. Consistency in temporal scope is also critical: if $R_{n}$ and $L_{n}$ are estimated from wildfires encountering treatments up to 15 y old, $p_{n}$ should reflect the same window. Ideally, the time window n of $R_{n}$, $L_{n}$, and $p_{n}$ should span the full effective lifespan of the treatment to capture its complete emissions benefit, not just the early period when it is most effective.

Although prescribed fire is unlikely to reduce net PM_2.5_ emissions in most places globally under current conditions, it may still lower population exposure to smoke. Exposure depends on transport and dispersion, which differ markedly between prescribed fires and wildfires (37): intense wildfires generate higher plumes that more often reach large population centers (95), whereas prescribed fire smoke can be managed under favorable meteorological conditions that limit population exposure (6, 37, 96). Wildfires—particularly those in the wildland–urban interface—are also more likely to burn structures (4), producing smoke that can be more toxic than smoke from prescribed fires (35, 97). Additionally, because prescribed fires are planned and their risks can be communicated in advance, smoke exposure may be more predictable and allow for more effective protective behaviors in communities, such as using portable air cleaners (98, 99). Thus, even if prescribed fire increases net emissions, safer and more predictable dispersion could still reduce health risks. Alternatively, because prescribed fires tend to be conducted close to population centers, they may incur higher health costs per unit of emissions than wildfire (58). Across global median conditions and the 73 sites analyzed, we find that prescribed fire smoke would need to have a 9 to 16-fold lower health impact per kg of emissions than wildfire smoke to offset increased emissions and yield a net health benefit (*SI Appendix*, *Supplemental Methods* and Fig. S5). While we know of no existing estimates with which to compare these values, it is conceivable that prescribed fire smoke has greatly reduced health impacts per kg of emissions, given the advantages described above (6, 96, 97). Determining the empirical ranges of these relative health impacts per kg of emissions is an important future research area, and will require integrating smoke transport modeling with spatially explicit estimates of downwind population exposure. Additionally, it is unclear whether the same total smoke exposure causes different health impacts when delivered as a single high-intensity event (e.g., a wildfire) or as lower-level exposures over time (e.g., prescribed fires) (7).

This work is not a critique of prescribed fire, nor does it suggest that prescribed fire is ineffective. Prescribed fires are unequivocally vital in achieving a wide range of ecological and cultural objectives (15–17). Many of these benefits occur regardless of whether wildfire subsequently interacts with the treated area, for example promoting valued habitat, reducing drought stress, increasing resistance to insects and disease, and providing “insurance” against extreme wildfire impacts to communities (17, 20). However, the emissions impacts of prescribed fire differ from other benefits: emissions carry an explicit cost in the form of treatment emissions, and the corresponding benefit does not occur passively but requires interaction with wildfire. Our findings highlight how prescribed fire can still be highly beneficial overall, even if it also has costs (46).

Prescribed fires clearly reduce severity and emissions in treated areas that later burn in wildfires (19, 22, 43, 64). However, estimating their net emissions impacts requires weighing treatment emissions against the likelihood that treatments will reduce subsequent wildfire emissions (Fig. 1*B*). Our results show that many studies overstate the net emissions benefits of prescribed fire and that true net reductions are generally limited to locations with very high encounter rates. We encourage future studies to include all emissions costs and benefits, and account for encounter probability. Prescribed fire is most likely to reduce net emissions in landscapes with high wildfire risk or in vegetation types where treatments substantially reduce subsequent wildfire burned area (i.e., high leverage). Regardless, prescribed fire remains essential for ecosystem health, infrastructure protection, and community and firefighter safety, among many other benefits (19, 20, 22, 81, 94). Understanding the net emissions impacts of prescribed fire can help practitioners design treatments that maximize these benefits while minimizing smoke exposure and health risk to humans.

## Materials and Methods

### Mathematical Framework.

We applied the mathematical framework described above (Eqs. **1**–**8**) to evaluate how prescribed fire affects net PM_2.5_ emissions change ($\Delta_{E}$) across a broad range of parameters: encounter probability ($p_{n}$), treatment emissions (T) expected proportion reduction within treated areas ($R_{n}$), and expected leverage of encountered treated areas ($L_{n}$). Because $p_{n}$, $R_{n}$, and $L_{n}$ depend on the chosen time window *n*, all three must be estimated for the same duration (e.g., 10 y). We explored parameter combinations spanning their plausible ranges: $p_{n}$ ∈ [0, 1], T ∈ [0, 1], $R_{n}$ ∈ [0, 1], and $L_{n}$ ∈ [−2, 4]. Although leverage is theoretically unbounded at very low proportions of the landscape treated (i.e., one hectare of treated area could prevent or promote wildfire burning across any number of hectares), the chosen range reflects empirically supported values (29). For each parameter set, we evaluated $\Delta_{E}$ directly from Eq. **5**, and visualized results (Fig. 2 and *SI Appendix*, Fig. S1) to illustrate the sensitivity of net emissions to variation in each parameter. As a supplemental analysis, we extended the mathematical framework to explore health impacts arising from PM_2.5_ emissions, implicitly accounting for emissions dispersion and human population exposure (*SI Appendix*, *Supplemental Materials*).

### Empirical Parameter Estimates.

We quantified empirical ranges of the four parameters ($p_{n}$, T, $R_{n}$, and $L_{n}$) using globally available datasets and published estimates. To estimate encounter probability ($p_{n}$), we used 10-y encounter rates (i.e., $p_{10-year}$) for National Forests (n = 108) in the United States from 2004–2023 (67). For a global estimate, we estimated encounter probabilities by calculating 10-y burn probabilities from monthly gridded burned area data from 1980–2020 at 0.5° resolution (72). To characterize 10-y burn probabilities where prescribed fire occurs, we extracted values at actual prescribed burn locations from the GlobalRx dataset (73), which contains 204,517 records from 1979–2023 across 16 countries.

Global data on treatment emissions (T), wildfire emission reductions ($R_{n}$), and leverage ($L_{n}$) are limited. To estimate empirical ranges of treatment emissions (T), we used reported values from 24 sites in our meta-analysis that estimated this value (*SI Appendix*, Table S1), as well as two other studies (56, 59). Additionally, we used Environmental Protection Agency state-level emissions inventory data from the United States (74) to calculate the ratio of average prescribed fire per-unit-area emissions to average wildfire per-unit area emissions for each state (n = 50). To estimate wildfire emission reductions ($R_{n}$), we used a meta-analysis of carbon emissions from treated and untreated forest stands in the western United States (64). Even though this source reported carbon emissions, the proportional reduction is exactly applicable to other emission species (e.g., PM_2.5_), since it reflects the reduction in fuel combusted under wildfire conditions, rather than differences in emission factors (90, 97). To estimate leverage ($L_{n}$) for encountered prescribed fire treatments, we used reported values of net leverage (i.e., wildfire area prevented minus wildfire area promoted) from a study modeling mechanical treatments and prescribed fires across 14 large U.S. wildfires (29). We also incorporated landscape-level leverage estimates (i.e., the total wildfire area prevented across a landscape divided by the total amount of treated area) from two additional analyses. The first (28) reports landscape-level leverage from both prescribed fire and wildfire (likely overestimating leverage effects from prescribed fire alone) across variable time windows between 1 and 8 y from six sites in Australia, South Africa, the United States, Canada, Portugal, and Spain. The second (30), from Portugal, reports landscape-level leverage from prescribed fire alone, within the previous 5 y. For use in our framework, we convert landscape leverage to conditional leverage (i.e., wildfire area prevented per unit of encountered prescribed fire area) by dividing the reported landscape leverage by the estimated encounter probability of treated area on each landscape. For example, if ten 1-ha prescribed fires collectively prevent 2 ha of wildfire, but only one treatment is actually encountered by wildfire, then 1 ha of encountered prescribed fire avoids 2 ha of wildfire.

### Previous Studies on Net Emissions Change.

We compiled studies reporting net emissions change (PM_2.5_ or CO_2_) from prescribed fires with sufficient data to estimate all four framework parameters. This included four studies (40, 41, 53, 75) from the review by Hunter and Robles (22), and seven recent analyses (23, 25, 38, 42, 43, 76, 77). Some studies (26, 27, 44, 45, 47–52, 56, 57) were not included in the reanalysis because parameters could not be extracted or inferred from the published data. For each included study, we extracted or derived the four parameters in our framework ($p_{n}$, T, $R_{n}$, and $L_{n}$).

We re-estimated parameter values for any parameter that was outside of plausible, empirical ranges. For studies that assumed encounter probabilities of 1, or close to 1 (i.e., ref. 43), we re-estimated encounter probabilities using empirical data. For studies in the United States (23, 25, 38, 40, 43, 77), we used 10-y treatment encounter rates from national forests (67) in the respective geography. For other studies, we used average 10-y wildfire probabilities (72) from each respective geography, or reported burn probability if included in the paper. For one study that omitted treatment emissions when evaluating prescribed fire across California (23), we re-estimated this parameter using California treatment emissions from the Environmental Protection Agency’s state-level emissions inventory (74). For two studies that assumed complete emissions reductions as a result of prescribed fire (i.e., $R_{n}$ = 1; 38, 40), we used the average proportion reduction from ref. 64 as described above. Finally, several studies (e.g., refs. 25 and 41) assumed leverage values far above empirically supported ranges (28–30), for example that every unit of prescribed fire prevents ~five units of wildfire across large regions (Europe and the United States). For these studies, we re-estimated leverage using the median empirical value from refs. 28–30. Re-estimated parameter values are shown in *SI Appendix*, Table S1. Using these updated parameters, we re-estimated net emissions change for all studies.

We conducted all analyses in R (100). R code and source data to reproduce all our results and figures are provided in a public dataset at https://zenodo.org/records/18318930 (101).

## Supplementary Material

Appendix 01 (PDF)

## Data Availability

Code and data have been deposited in Zenodo (https://doi.org/10.5281/zenodo.18318930) (101).
