# Supplementary material for: Prescribed fire is unlikely to reduce net PM2.5 emissions in most locations
Source: Proc Natl Acad Sci U S A. 2026 Jun 29;123(28):e2613722123. doi: 10.1073/pnas.2613722123 (PMC13367821; doi:10.1073/pnas.2613722123)
Supplement: Supplementary file 1 — Appendix 01 (PDF) [file pnas.2613722123.sapp.pdf]

PNAS

Supporting Information for

Prescribed fire is unlikely to reduce net $PM_{2.5}$ emissions in most locations

Mark R. Kreider
Shawn P. Urbanski
Joseph Fargione

Corresponding author: Mark Kreider
Email: mark.kreider@usda.gov

This PDF file includes:

Supplemental Methods
Figures S1 to S8
Tables S1
SI References

Supplemental Methods

Deriving net emissions change equation from long-term fire dynamics

Instead of deriving the framework from probabilistic encounter rates over relatively short timeframes (i.e., ~one to two decades, or the effective duration of a treatment), the same net emissions change equation (main manuscript Equation 6) can also be derived from longer-term timeframes.

Consider a landscape with an average wildfire return interval $\emptyset_{wildfire}$, in years. For example, if $\emptyset_{wildfire}$ = 100 years, then the cumulative area burned by a wildfire over a 100-year period is, on average, equal to the area of the landscape (though this does not necessarily mean that every point burns). Over any time window W (in years), the expected cumulative fraction burned ($Z_{wildfire}$) is:

$$Z_{wildfire} = \frac{W}{\emptyset_{wildfire}}$$

Equation S1

Note that $Z_{wildfire}$ can exceed 1 when $W > \emptyset_{wildfire}$, which simply means that the cumulative area burned over that period is larger than the landscape area (i.e., some or all of the landscape has burned multiple times).

In a scenario with only wildfire (i.e., no prescribed fire), the total wildland fire emissions over time window W ($E_{NoRx\,|\,W}$) are equal to the cumulative fraction burned ($Z_{wildfire}$) multiplied by the landscape area (A) multiplied by the average per-unit-area wildfire emissions (y):

$$E_{NoRx\,|\,W} = Z_{wildfire} \cdot A \cdot y$$

Equation S2

In a scenario with the addition of prescribed fire, we assume that prescribed fires are effective for a fixed lifespan of n years, after which the location is re-treated with prescribed fire. In other words, for the proportion Q of the landscape that is maintained with prescribed fire, the prescribed fire return interval ($\emptyset_{Rx}$) is equal to n. Thus, over time window W, the cumulative fraction of the total landscape treated with prescribed fire is:

$$Z_{Rx} = Q \cdot \frac{W}{\emptyset_{Rx}}$$

Equation S3

In the scenario with prescribed fire, the total wildland fire emissions over time window W ($E_{Rx\,|\,W}$) are the sum of two components. First, the emissions from the prescribed fires themselves are equal to the cumulative fraction burned with prescribed fire (Z_{Rx}) multiplied by the landscape area (A) multiplied by the average per-unit-area prescribed fire emissions (x). Second, the emissions from wildfires that still occur are calculated as follows. On the treated portion of the landscape (Q), subsequent wildfire emissions equal the expected wildfire emissions conditional on burning ($Z_{wildfire} \cdot A \cdot y$), reduced by the proportional reduction in wildfire emissions due to lower fuel loads (R_n) and the avoided wildfire emissions outside of the treated areas from leverage (L_n; average units of wildfire avoided for each unit of encountered prescribed fire). With avoided wildfire due to leverage already accounted for, the remaining untreated portion of the landscape ($1 - Q$) has emissions of $Z_{wildfire} \cdot A \cdot y$. Thus, the total emissions under the prescribed fire scenario over time window W ($E_{Rx\,|\,W}$) are equal to:

$$E_{Rx\,|\,W} = Z_{Rx} \cdot A \cdot x + Q \cdot Z_{wildfire} \cdot A \cdot y \cdot (1 - R_n - L_n) + (1 - Q)(Z_{wildfire} \cdot A \cdot y)$$

Equation S4

For time window W, we can calculate the proportional change in emissions between the scenarios with and without prescribed fire (Δ_{E_W}) as:

$$\Delta_{E_W} = \frac{E_{Rx\,|\,W} - E_{NoRx\,|\,W}}{E_{NoRx\,|\,W}}$$

Equation S5

Evaluating Equation S5 with Equations S1–S4 yields the simplified equation:

$$\Delta_{E_W} = Q\left(\frac{x}{y} \cdot \frac{\emptyset_{wildfire}}{\emptyset_{Rx}} - (R_n + L_n)\right)$$

Equation S6

Defining treatment emissions (T) as a proportion of untreated wildfire emissions $\left(T = \frac{x}{y}\right)$ allows Δ_W to be expressed as:

$$\Delta_{E_W} = Q\left(T \cdot \frac{\emptyset_{wildfire}}{\emptyset_{Rx}} - (R_n + L_n)\right)$$

Equation S7

Equation S7 shows that the proportional change in emissions from prescribed fire depends on the ratio of wildfire frequency to prescribed fire frequency (i.e., prescribed fire longevity), or $\frac{\emptyset_{wildfire}}{\emptyset_{Rx}}$. Prescribed fire will cause larger reductions in emissions when $\frac{\emptyset_{wildfire}}{\emptyset_{Rx}}$ is minimized, for example if prescribed fires can remain effective for longer ($\emptyset_{Rx}$ increases while R_n and L_n remain unchanged), or if wildfires become more frequent ($\emptyset_{wildfire}$ decreases).

If wildfire occurrence at a point follows a Poisson process with a constant annual hazard, the probability of experiencing at least one wildfire within an n-year period (p_n) is:

$$p_n = 1 - e^{-\frac{n}{\emptyset_{wildfire}}}$$

Equation S8

Solving for $\emptyset_{wildfire}$ gives:

$$\emptyset_{wildfire} = -\frac{n}{\ln{(1 - p_n)}}$$

Equation S9

When there is a low probability of wildfire (i.e., small values of p_n), the logarithmic term can be approximated using $\ln(1 - p_n) \approx -p_n$, yielding:

$$\emptyset_{wildfire} \approx \frac{n}{p_n}$$

Equation S10

This linear approximation is accurate when wildfire occurrence is rare (e.g. $p_n < 0.15$; Figure S8)—almost all cases with at least one fire have exactly one fire in the window. As p_n increases, the likelihood of multiple fires within the n-year window becomes non-negligible, so the linear approximation $\emptyset_{wildfire} \approx \frac{n}{p_n}$ diverges from the exact expression $\emptyset_{wildfire} = -\frac{n}{\ln{(1-p_n)}}$. However, in many parts of the world, treatment encounter probability is relatively rare (e.g., average 10-year encounter rate of ~7% across the contiguous U.S.; (1)).

The return interval of prescribed fire ($\emptyset_{Rx}$) was defined earlier as the average effective lifespan of a treatment (n, in years); $\emptyset_{Rx} = n$. At low fire probabilities, since $\emptyset_{wildfire} \approx \frac{n}{p_n}$, the fraction $\frac{\emptyset_{wildfire}}{\emptyset_{Rx}}$ can be approximated as:

$$\frac{\emptyset_{wildfire}}{\emptyset_{Rx}} \approx \frac{\frac{n}{p_n}}{n} = \frac{1}{p_n}$$

Equation S11

Thus, at low fire probabilities (i.e., small values of p_n), Equation S7 can be approximated as:

$$\Delta_{E_W} \approx Q\left(\frac{T}{p_n} - (R_n + L_n)\right)$$

Equation S12

Note that Equation S12 is equivalent to Equation 7 in the Main Text. This equivalency holds true provided two conditions are met: 1) p_n is relatively small, and 2) the time window n chosen for p_n is the same as the effective lifespan of prescribed fire treatments (i.e., $n = \emptyset_{Rx}$).

Extending framework to health impacts of emissions

While it is useful to understand how prescribed fire impacts emissions, the ultimate impact of $PM_{2.5}$ emissions on human health depends on how emissions are dispersed, and the resulting concentrations in locations where people live (2, 3). In theory, then, the change in $PM_{2.5}$ health impacts as a result of prescribed fire (Δ_{health}) is the proportional change in a given health impact under the scenario with prescribed burning (I_{Rx}) relative to a scenario with no prescribed burning (I_{NoRx}):

$$\Delta_{health} = \frac{I_{Rx} - I_{NoRx}}{I_{NoRx}}$$

Equation S13

Without prescribed fire, the expected per-unit-area health impact from emissions across a landscape is the expected emissions ($p_n y$) multiplied by some coefficient of health impact per kilogram of $PM_{2.5}$ emissions from wildfire (H_y).

$$I_{NoRx} = p_n y \cdot H_y$$

Equation S14

Similarly, in the scenario with Q proportion of the landscape treated with prescribed fire, the expected health impact can be derived from the equation for expected emissions (i.e., main text Equation 3) where prescribed fire emissions are multiplied by a coefficient of health impact per kilogram of $PM_{2.5}$ emissions from prescribed fire (H_x), and wildfire emissions are similarly multiplied by the wildfire emissions health impact coefficient (H_y).

$$I_{Rx} = Q \cdot x \cdot H_x + Q \cdot p_n y(1 - R_n - L_n) \cdot H_y + (Q - 1)(p_n y) \cdot H_y$$

Equation S15

Because prescribed fires are often conducted under favorable meteorological conditions that attempt to minimize smoke exposure to large population centers (4–6), the health impact of 1 kg of $PM_{2.5}$ emissions may be lower for prescribed fire emissions relative to wildfire emissions; if so, $H_x < H_y$. We define the proportional relationship of these two values as H, or the relative health impact of prescribed fire per unit of emitted $PM_{2.5}$:

$$H = \frac{H_x}{H_y}$$

Equation S16

Evaluating Equation S13 with Equations S14–S16, we show that the change in $PM_{2.5}$ health impacts as a result of prescribed fire (Δ_{health}) is equal to:

$$\Delta_{health} = Q\left(H \cdot \frac{T}{p_n} - (R_n + L_n)\right)$$

Equation S17

While, to our knowledge, there are no existing empirical estimates of the value of H, Equation S17 can be re-written to find the value of H that would be required for prescribed fire to reduce overall health impacts, which we denote as H^* (i.e., the value of H that makes $\Delta_{health} = 0$).

$$H^* = \frac{p_n(R_n + L_n)}{T}$$

Equation S18

Equation S18 is identical to the ratio of avoided emissions to added emissions (Equation 8 in the main text). In other words, when prescribed fire adds more emissions than it avoids, the per-kilogram health impact must be lower by exactly this ratio to offset the added emissions. We evaluate the value of H^* across plausible ranges of parameters (Figure S5). At median values of global parameters (p_n = 0.07, T = 0.48, R_n = 0.56, L_n = 0.27), H^* = 0.115. Thus, at median parameter values, the per-kg-emissions health impacts of prescribed fire would have to be 8.7 times lower than wildfire per-kg-emissions health impacts for prescribed fire to lead to a health benefit.

Supplemental Figures

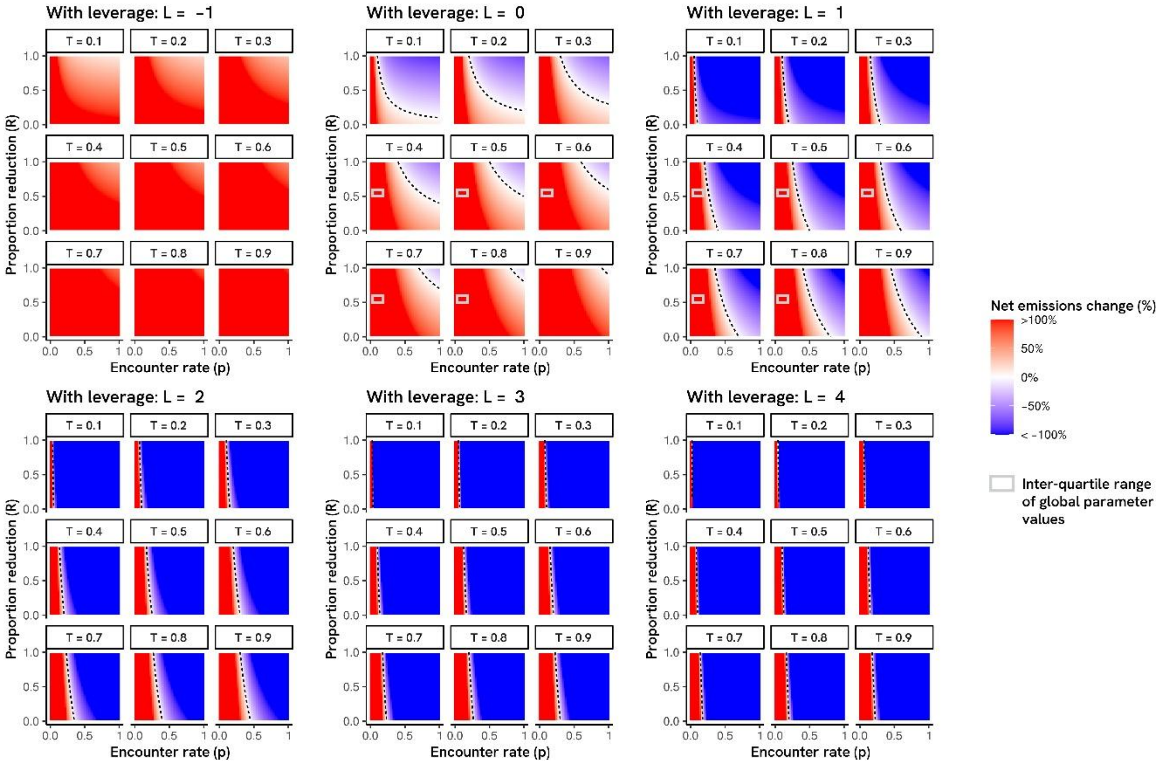

Figure S1. Net emissions change across a full range of parameters. A) Net emissions change across possible ranges of p_n (treatment encounter rate), R_n (proportional reduction in wildfire emissions as a result of being treated), L_n (leverage; wildfire area prevented per unit of encountered treated area), T (treatment emissions; the ratio of prescribed fire emissions to untreated wildfire emissions per unit area). The dashed line depicts the point at which net emissions change is 0. Grey rectangles show the parameter space which falls within the inter-quantile range (25%–75%) from empirically estimated values of p_n, R_n, L_n, T. Net emissions change greater than 1 (i.e., >100%) are all depicted as the same shade of red.

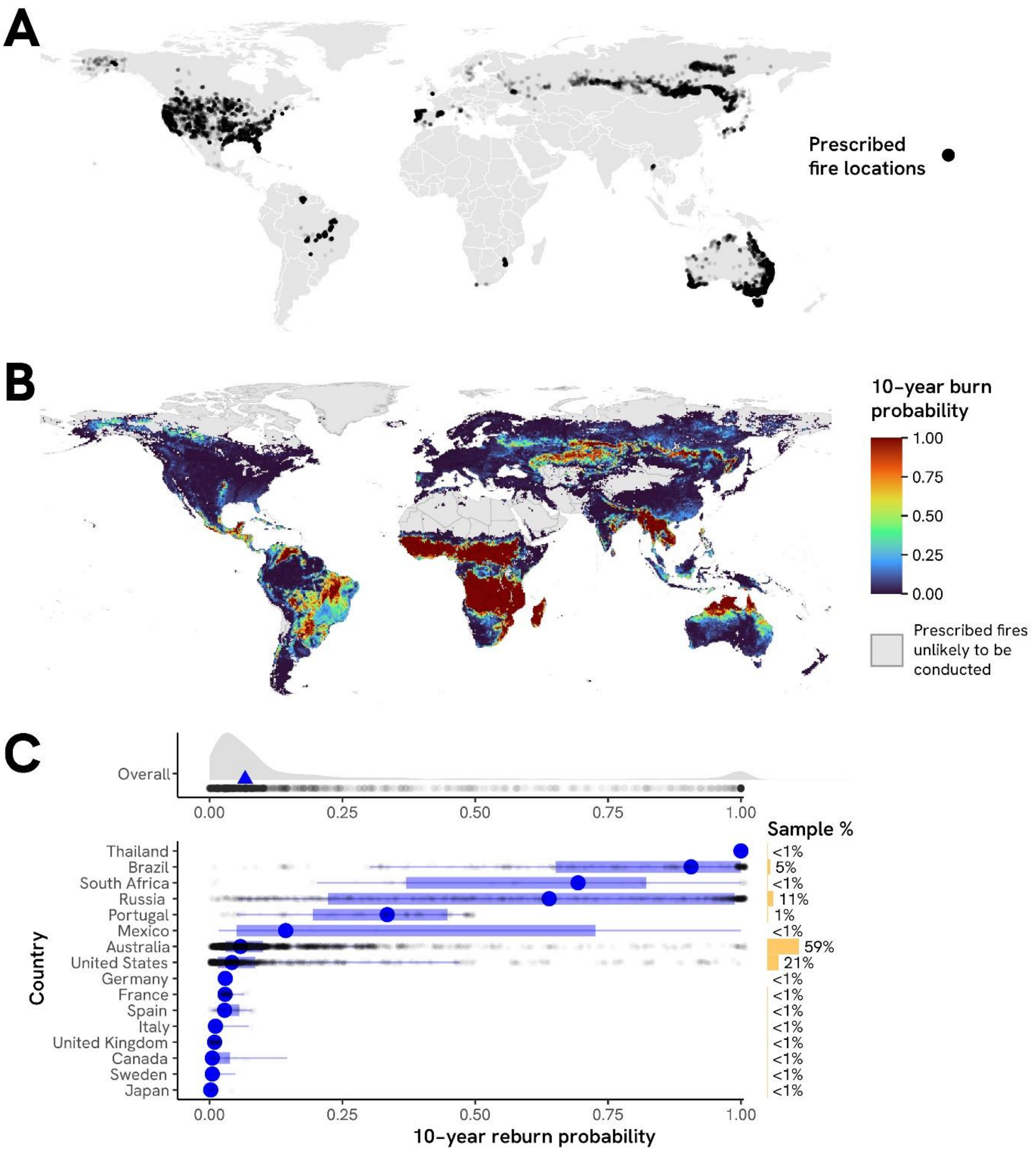

Figure S2. 10-year burn probability of global prescribed fire treatments. A) Locations of global prescribed fires from GlobalRx dataset (7); a random sample of 50,000 treatment centroids are shown. Note that GlobalRx only includes prescribed fire records from 16 countries. B) Global 10-year burn probability, averaged across all 10-year windows beginning in 1980 to 2011 (8). C) 10-year burn probability by country. Transparent points depict individual values for prescribed burn centroids, while lines depict variability in the sample (thin line is central 90% quantile [5-95%]; thick line is interquartile range [25-75%]; and the blue point is the median value). The overall distribution is shown above with the blue triangle depicting the overall median value.

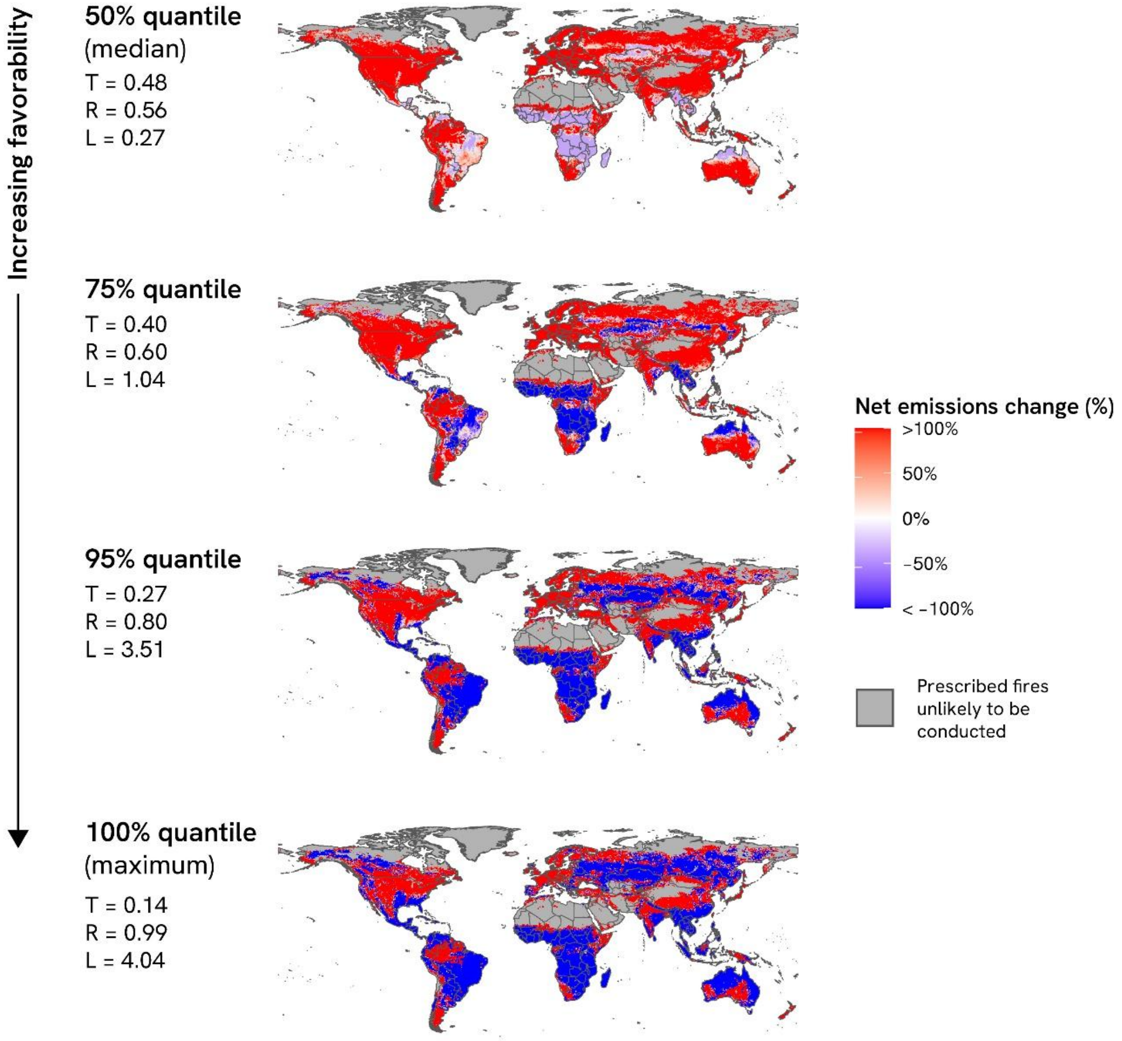

Figure S3. Net emissions change under increasingly favorable parameters of T (treatment emissions; the ratio of prescribed fire emissions to untreated wildfire emissions per unit area), R_n (proportional reduction in wildfire emissions as a result of being treated), and L_n (leverage; wildfire area prevented per unit of encountered treated area). Net emissions change is calculated using Equation 5 from the main text, where p_n is equal to the average 10-year burn probability globally (8). Values of T, R_n, and L_n come from distributions of parameter values as shown in Figure 2B. Values below -1 have been capped at -1; values above 3 have been capped at 3. Net emissions change is only shown for pixels of vegetation types where prescribed burning is commonly conducted; other pixels are shown as grey. Note that parameters likely cannot be simultaneously maximized (e.g., increases in R_n and L_n would likely only be possible with more fuel removed during a treatment, hence an increase in T; Figure 1B).

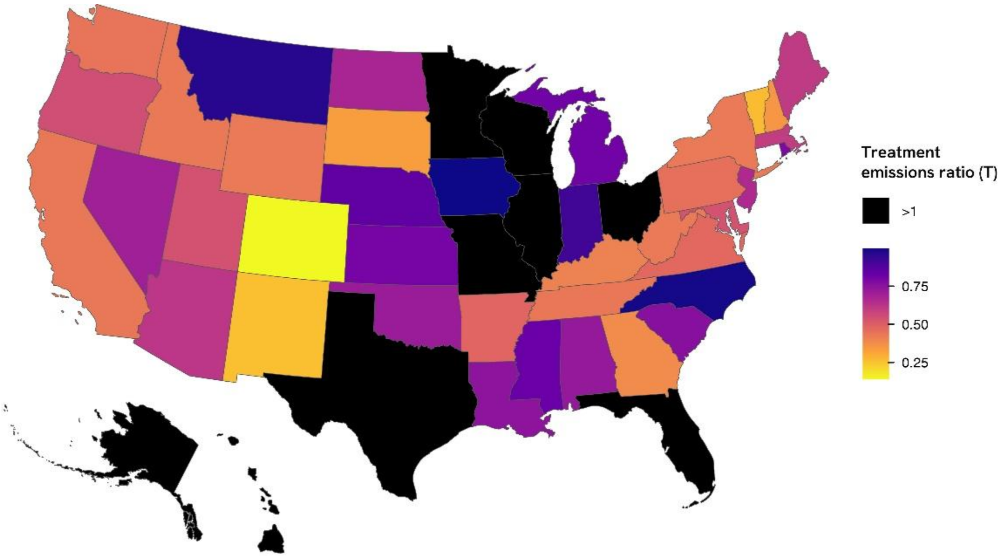

Figure S4. Average prescribed fire emissions proportion (T; i.e., relative to untreated wildfire emissions per unit area) for U.S. states, calculated from EPA state-level 2020 emissions inventory data (9). Geometries where prescribed fire per-unit-area emissions were greater than per-unit wildfire emissions are capped at 1 for visualization.

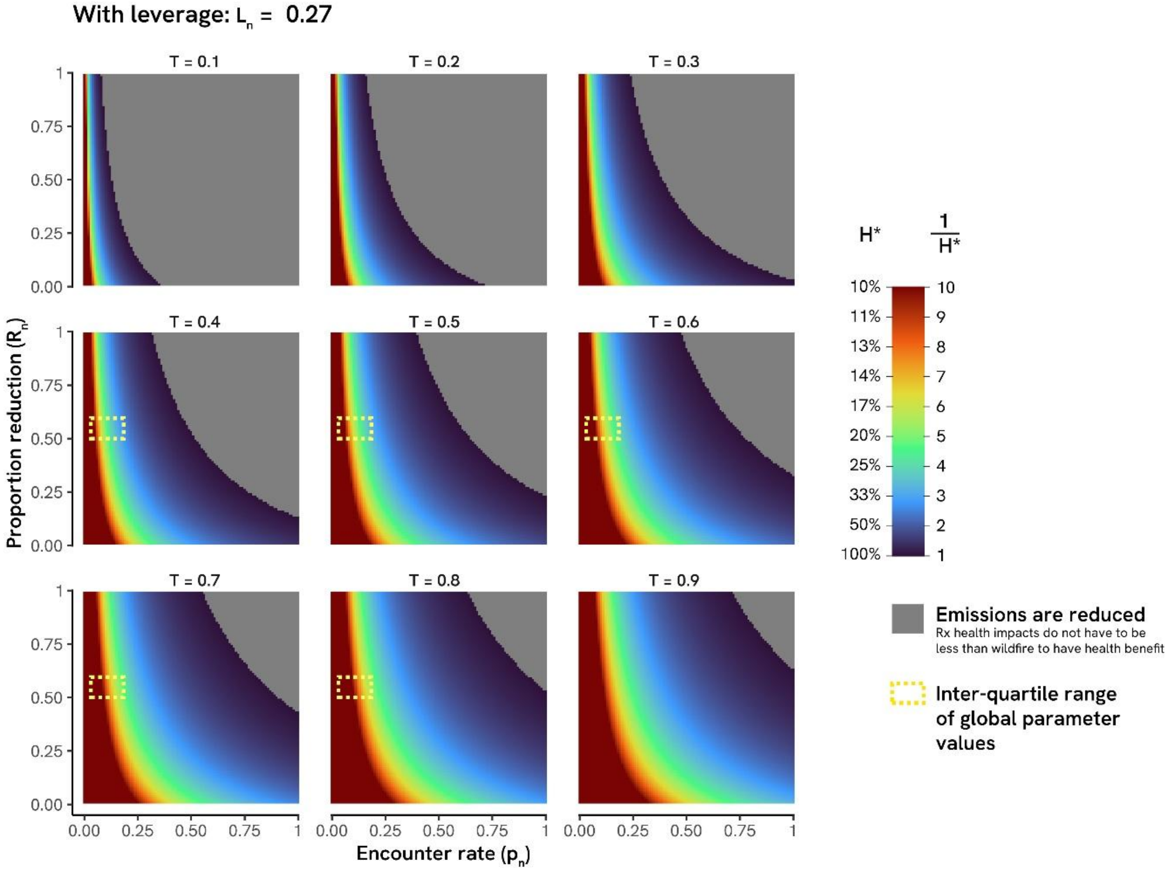

Figure S5. Required values of H^* (the proportional health impact per unit of emissions for prescribed fire relative to wildfire) such that a net health benefit could still be achieved. The reciprocal of H^* is also shown $\left(\frac{1}{H^*}\right)$, representing how many more times less of an impact prescribed fire emissions would need to have relative to wildfire emissions. Values of H^* are not shown in regions of parameter space where net emissions reductions occur, as net health benefits would still be achieved even if health impacts were the same per unit of emissions between prescribed fire and wildfire. Yellow dashed rectangles show the parameter space which falls within the inter-quantile range (25%–75%) from empirically estimated values of p_n, R_n, T. L_n is held at its median value (0.27).

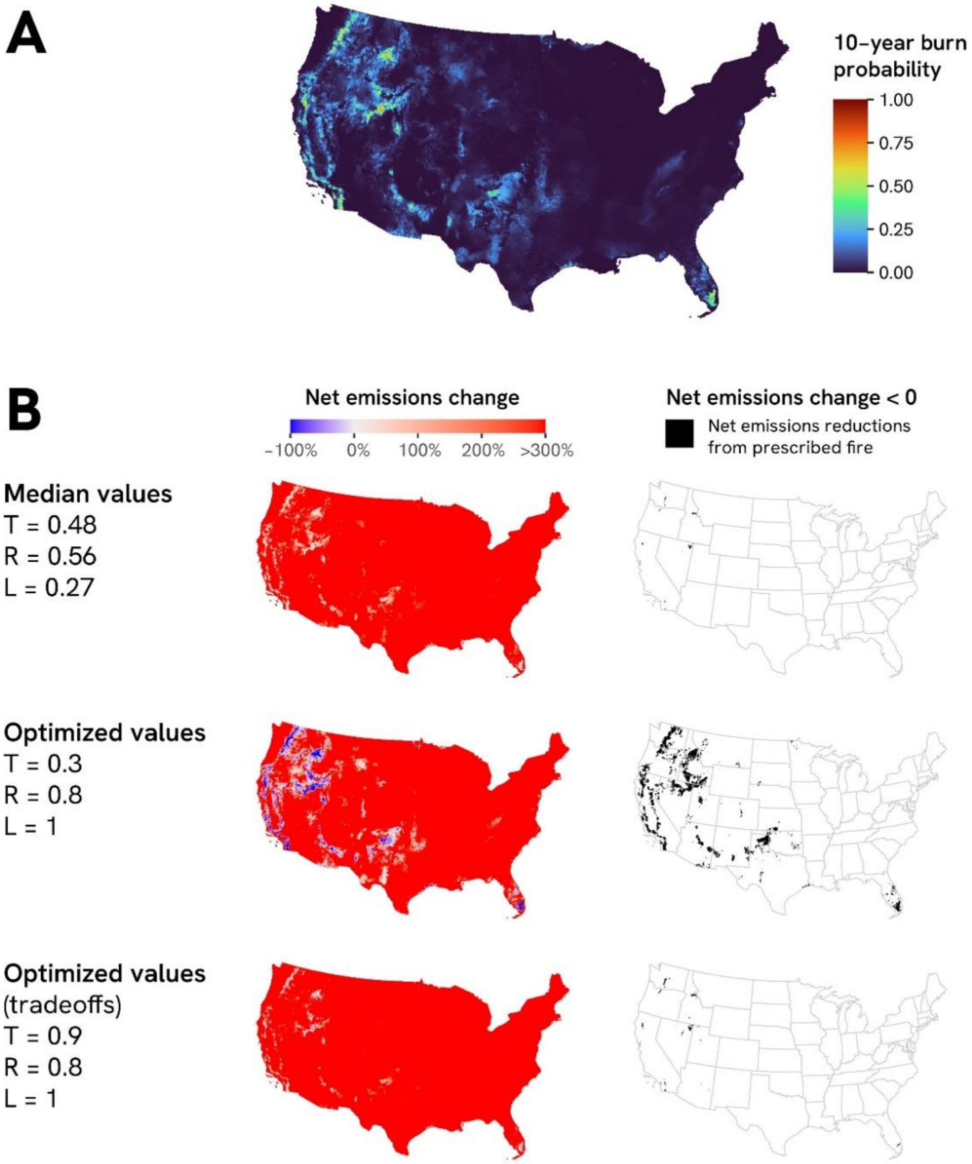

Figure S6. Net emissions change framework applied to the contiguous U.S. A) 10-year burn probability, calculated from annual burn probability modeled output from FSim, a spatially explicit large-fire simulation model calibrated to historical fire activity and weather (10). B) Net emissions change (left column) and locations of net emissions reductions (right column) under different values of T (treatment emissions; the ratio of prescribed fire emissions to untreated wildfire emissions per unit area), R_n (proportional reduction in wildfire emissions as a result of being treated), and L_n (leverage; wildfire area prevented per unit of encountered treated area). Net emissions change is calculated using Equation 6, where p_n is equal to the average 10-year burn probability from panel A. Top: net emissions change under median global values of T, R, and L; Middle: net emissions change under hypothetical optimized values of T, R_n, and L_n for the contiguous U.S., without accounting for inherent tradeoffs in parameters; Bottom: net emissions change if inherent tradeoffs are accounted for (e.g., increased treatment effectiveness [high R_n and L_n] is likely only possible with high levels of fuel removed during treatment, resulting in high treatment emissions). Values below -1 or above 3 have been capped at -1 and 3, respectively.

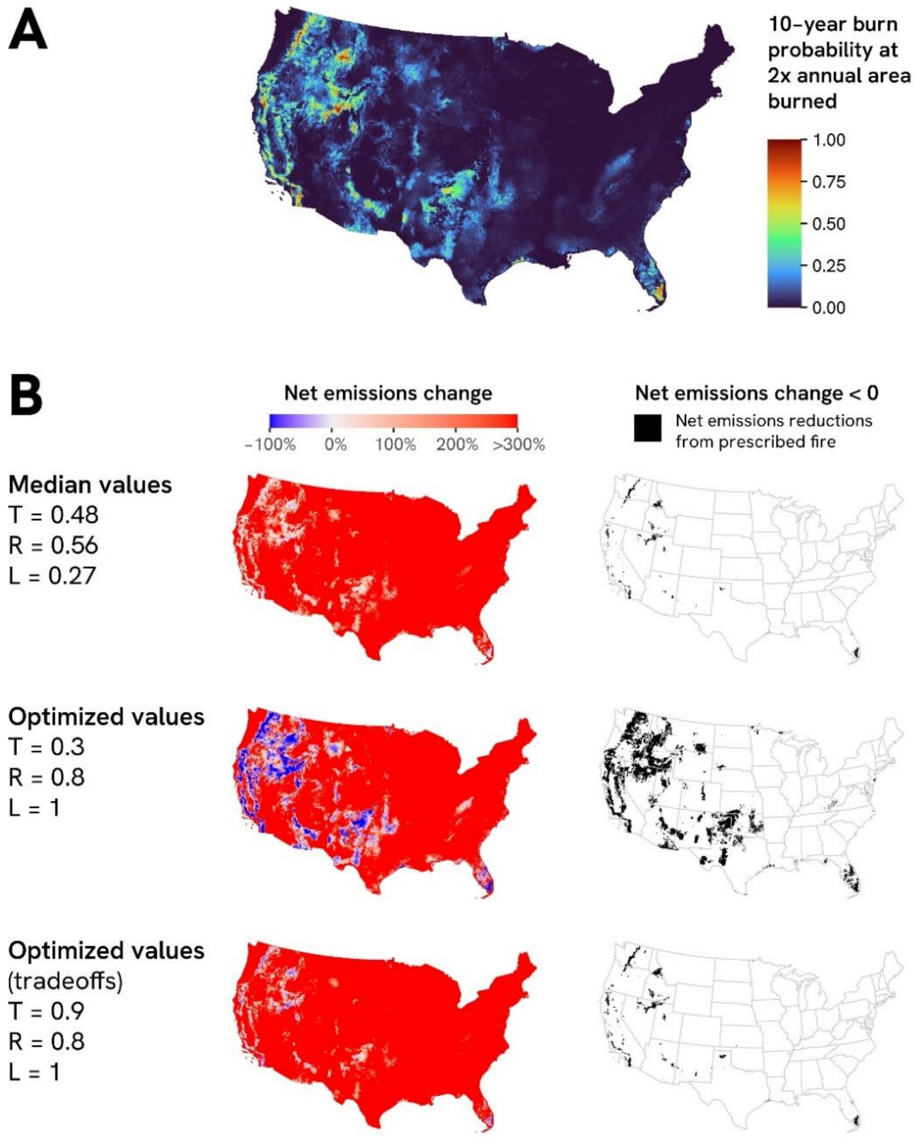

Figure S7. Net emissions change of contiguous U.S. under hypothetical increase in area burned A) 10-year burn probability, at a hypothetical two-times higher area burned, relative to annual probability modeled output from FSim, a spatially explicit large-fire simulation model calibrated to historical fire activity and weather (10). B) Net emissions change (left column) and locations of net emissions reductions (right column) under different values of T (treatment emissions; the ratio of prescribed fire emissions to untreated wildfire emissions per unit area), R_n (proportional reduction in wildfire emissions as a result of being treated), and L_n (leverage; wildfire area prevented per unit of encountered treated area). Net emissions change is calculated using Equation 6, where p_n is equal to the average 10-year burn probability from panel A. Top: net emissions change under median global values of T, R_n, and L_n; Middle: net emissions change under hypothetical optimized values of T, R_n, and L_n for the contiguous U.S., without accounting for inherent tradeoffs in parameters; Bottom: net emissions change if inherent tradeoffs are accounted for (e.g., increased treatment effectiveness [high R_n and L_n] is likely only possible with high levels of fuel removed during treatment, resulting in high treatment emissions). Values below -1 or above 3 have been capped at -1 and 3, respectively.

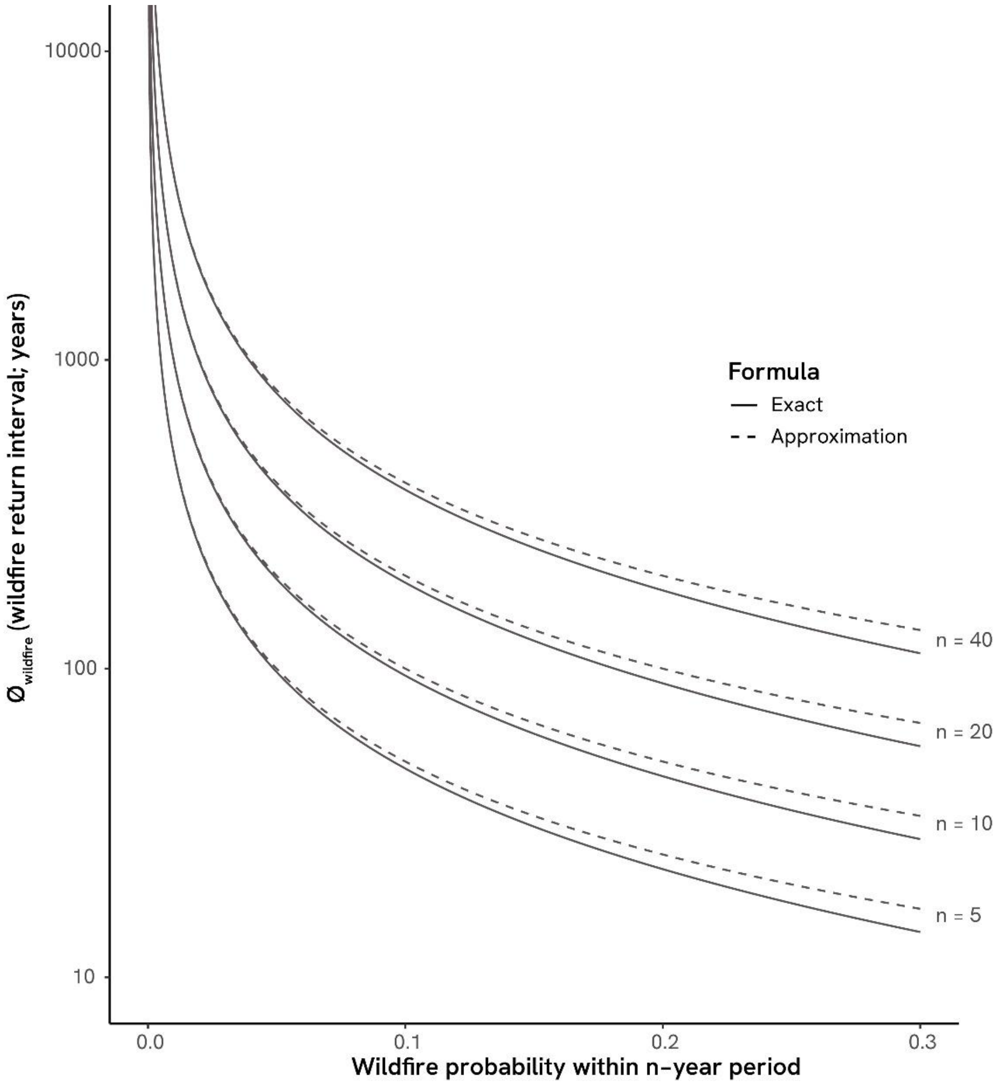

Figure S8. Difference between exact $\left(-\frac{n}{\ln(1-p_n)}\right)$ and approximated $\left(\frac{n}{p_n}\right)$ values of wildfire return interval ($\emptyset_{wildfire}$) across values of p_n and n.

Supplemental Tables

Table S1. Reported and re-estimated parameters of studies examining net emissions change from treatments. Note that some studies (11–22) could not be included in the re-analysis because parameters could not be extracted or inferred from the published data. If reported parameters were changed (i.e., an assumed encounter probability p_n of 1, changed to an empirical estimate), the updated parameter value is stated following the reported parameter, separated by the symbol "|". Unless otherwise reported, estimates of studies refer to the entire country. Parameter values are rounded to two digits in this table: re-estimated emissions change percentages may be slightly different than the value calculated from rounded parameter values. Values of p_n are >0 even though some rounded values may appear as 0.

			Parameters [original | **re-estimated**]					Net emissions change (%)	
Study	Emission species	Location	T	p_n	R_n	L_n	Q	Reported	Re-estimated
Narayan et al. 2007 (23)	CO_2	Albania	0.39	1 | **0.09**	0 | **0.56**	5 | **0.27**	0.1	-46%	37%
		Algeria	0.39	1 | **0.04**	0 | **0.56**	5 | **0.27**	0.1	-46%	84%
		Austria	0.39	1 | **0.00**	0 | **0.56**	5 | **0.27**	0.1	-46%	930%
		Belarus	0.39	1 | **0.06**	0 | **0.56**	5 | **0.27**	0.1	-46%	58%
		Belgium	0.39	1 | **0.00**	0 | **0.56**	5 | **0.27**	0.1	-46%	802%
		Bulgaria	0.39	1 | **0.05**	0 | **0.56**	5 | **0.27**	0.1	-46%	72%
		Croatia	0.39	1 | **0.04**	0 | **0.56**	5 | **0.27**	0.1	-46%	83%
		Cyprus	0.39	1 | **0.01**	0 | **0.56**	5 | **0.27**	0.1	-46%	352%
		Czech Republic	0.39	1 | **0.01**	0 | **0.56**	5 | **0.27**	0.1	-46%	342%
		Denmark	0.39	1 | **0.00**	0 | **0.56**	5 | **0.27**	0.1	-46%	1639%
		Estonia	0.39	1 | **0.03**	0 | **0.56**	5 | **0.27**	0.1	-46%	129%
		Finland	0.39	1 | **0.01**	0 | **0.56**	5 | **0.27**	0.1	-46%	256%
		France	0.39	1 | **0.01**	0 | **0.56**	5 | **0.27**	0.1	-46%	398%
		Germany	0.39	1 | **0.01**	0 | **0.56**	5 | **0.27**	0.1	-46%	657%
		Greece	0.39	1 | **0.02**	0 | **0.56**	5 | **0.27**	0.1	-46%	148%
		Israel	0.39	1 | **0.02**	0 | **0.56**	5 | **0.27**	0.1	-46%	194%
		Italy	0.39	1 | **0.03**	0 | **0.56**	5 | **0.27**	0.1	-46%	134%
		Latvia	0.39	1 | **0.04**	0 | **0.56**	5 | **0.27**	0.1	-46%	96%
		Lithuania	0.39	1 | **0.03**	0 | **0.56**	5 | **0.27**	0.1	-46%	125%
		Luxembourg	0.39	1 | **0.00**	0 | **0.56**	5 | **0.27**	0.1	-46%	1511%
		Morocco	0.39	1 | **0.01**	0 | **0.56**	5 | **0.27**	0.1	-46%	664%
		Netherlands	0.39	1 | **0.00**	0 | **0.56**	5 | **0.27**	0.1	-46%	995%
		Norway	0.39	1 | **0.00**	0 | **0.56**	5 | **0.27**	0.1	-46%	1193%
		Poland	0.39	1 | **0.01**	0 | **0.56**	5 | **0.27**	0.1	-46%	269%
		Portugal	0.39	1 | **0.33**	0 | **0.56**	5 | **0.27**	0.1	-46%	3%
		Slovakia	0.39	1 | **0.01**	0 | **0.56**	5 | **0.27**	0.1	-46%	276%
		Slovenia	0.39	1 | **0.01**	0 | **0.56**	5 | **0.27**	0.1	-46%	557%
		Spain	0.39	1 | **0.03**	0 | **0.56**	5 | **0.27**	0.1	-46%	104%
		Sweden	0.39	1 | **0.01**	0 | **0.56**	5 | **0.27**	0.1	-46%	341%
		Switzerland	0.39	1 | **0.00**	0 | **0.56**	5 | **0.27**	0.1	-46%	936%
		North Macedonia	0.39	1 | **0.08**	0 | **0.56**	5 | **0.27**	0.1	-46%	43%
		Turkey	0.39	1 | **0.01**	0 | **0.56**	5 | **0.27**	0.1	-46%	299%
		United Kingdom	0.39	1 | **0.01**	0 | **0.56**	5 | **0.27**	0.1	-46%	279%
Hurteau & North 2009 (24)	C	USA: site in California	0.30	0.20	0.54	0 | **0.27**	1	97%	70%
Wiedinmyer & Hurteau 2010 (25)	CO_2	USA: Arizona	0.48	1 | **0.22**	1 | **0.56**	0 | **0.27**	1	-52%	138%
		USA: California	0.40	1 | **0.14**	1 | **0.56**	0 | **0.27**	1	-60%	212%
		USA: Colorado	0.40	1 | **0.06**	1 | **0.56**	0 | **0.27**	1	-60%	633%
		USA: Idaho	0.38	1 | **0.13**	1 | **0.56**	0 | **0.27**	1	-62%	210%
		USA: Montana	0.37	1 | **0.05**	1 | **0.56**	0 | **0.27**	1	-63%	701%
		USA: Nevada	0.43	1 | **0.01**	1 | **0.56**	0 | **0.27**	1	-57%	3204%
		USA: New Mexico	0.63	1 | **0.07**	1 | **0.56**	0 | **0.27**	1	-37%	853%
		USA: Oregon	0.44	1 | **0.08**	1 | **0.56**	0 | **0.27**	1	-56%	476%
		USA: Utah	0.46	1 | **0.10**	1 | **0.56**	0 | **0.27**	1	-54%	369%
		USA: Washington	0.38	1 | **0.16**	1 | **0.56**	0 | **0.27**	1	-62%	152%
		USA: Wyoming	0.43	1 | **0.06**	1 | **0.56**	0 | **0.27**	1	-57%	586%
Vilén & Fernandes 2011 (26)	CO_2	France	0.38	1 | **0.01**	0 | **0.56**	1 | **0.27**	0.02	-1%	79%
		Greece	0.38	1 | **0.02**	0 | **0.56**	1 | **0.27**	0.02	-1%	29%
		Italy	0.38	1 | **0.03**	0 | **0.56**	1 | **0.27**	0.02	-1%	26%
		Portugal	0.38	1 | **0.33**	0 | **0.56**	1 | **0.27**	0.02	-1%	1%
		Spain	0.38	1 | **0.03**	0 | **0.56**	1 | **0.27**	0.02	-1%	20%
		France	0.38	1 | **0.01**	0 | **0.56**	1 | **0.27**	0.2	-12%	785%
		Greece	0.38	1 | **0.02**	0 | **0.56**	1 | **0.27**	0.2	-12%	292%
		Italy	0.38	1 | **0.03**	0 | **0.56**	1 | **0.27**	0.2	-12%	265%
		Portugal	0.38	1 | **0.33**	0 | **0.56**	1 | **0.27**	0.2	-12%	6%
		Spain	0.38	1 | **0.03**	0 | **0.56**	1 | **0.27**	0.2	-12%	205%
		France	0.38	1 | **0.01**	0 | **0.56**	3 | **0.27**	0.02	-5%	79%
		Greece	0.38	1 | **0.02**	0 | **0.56**	3 | **0.27**	0.02	-5%	29%
		Italy	0.38	1 | **0.03**	0 | **0.56**	3 | **0.27**	0.02	-5%	26%
		Portugal	0.38	1 | **0.33**	0 | **0.56**	3 | **0.27**	0.02	-5%	1%
		Spain	0.38	1 | **0.03**	0 | **0.56**	3 | **0.27**	0.02	-5%	20%
		France	0.38	1 | **0.01**	0 | **0.56**	3 | **0.27**	0.2	-52%	785%
		Greece	0.38	1 | **0.02**	0 | **0.56**	3 | **0.27**	0.2	-52%	292%
		Italy	0.38	1 | **0.03**	0 | **0.56**	3 | **0.27**	0.2	-52%	265%
		Portugal	0.38	1 | **0.33**	0 | **0.56**	3 | **0.27**	0.2	-52%	6%
		Spain	0.38	1 | **0.03**	0 | **0.56**	3 | **0.27**	0.2	-52%	205%
Defossé et al. 2011 (27)	CO_2	Argentina: site in Patagonia	0.35	1 | **0.06**	0 | **0.56**	2.5 | **0.27**	0.2	-43%	92%
Volkova et al. 2014 (28)	CO_2	Australia: site in Victoria	0.18	1 | **0.50**	0.60	0 | **0.27**	1	-43%	-52%
Burke et al. 2021 (29)	$PM_{2.5}$	USA: CONUS	0.50	1 | **0.09**	0 | **0.56**	1.8 | **0.27**	0.116	-15%	56%
		USA: CONUS	0.50	1 | **0.09**	0 | **0.56**	4.9| **0.27**	0.116	-51%	56%
Kelp et al. 2023 (30)	$PM_{2.5}$	USA: western US	0.00 | **0.43**	1 | **0.10**	0.5	0 | **0.27**	1	-50%	351%
Kiely et al. 2024 (31)	$PM_{2.5}$	USA: northern California	0.48	1 | **0.13**	1.0 | **0.56**	0 | **0.27**	1	-52%	288%
Kelp et al. 2025 (32)	$PM_{2.5}$	USA: California	0.17	0.80 | **0.14**	0.3	0 | **0.27**	1	-13%	64%
Li et al. 2025 (33)	$PM_{2.5}$	USA: site in Tennessee	0.52	1 | **0.04**	0.6	0 | **0.27**	1	-6%	1215%

Supplemental References:

1. J. L. Beidler, K. R. Baker, G. Pouliot, J. D. Sacks, Encountering Prescribed Fire: Characterizing the Intersection of Prescribed Fire and Wildfire in the CONUS. *ACS EST Air* **1**, 1687–1695 (2024).

2. A. Rosenberg, *et al.*, Health Impacts of Future Prescribed Fire Smoke: Considerations From an Exposure Scenario in California. *Earth's Future* **12**, e2023EF003778 (2024).

3. G. J. Williamson, D. M. J. S. Bowman, O. F. Price, S. B. Henderson, F. H. Johnston, A transdisciplinary approach to understanding the health effects of wildfire and prescribed fire smoke regimes. *Environ. Res. Lett.* **11**, 125009 (2016).

4. S. M. D'Evelyn, *et al.*, Wildfire, Smoke Exposure, Human Health, and Environmental Justice Need to be Integrated into Forest Restoration and Management. *Curr Envir Health Rpt* **9**, 366–385 (2022).

5. J. W. Long, L. W. Tarnay, M. P. North, Aligning smoke management with ecological and public health goals. *Journal of Forestry. 116(1): 76-86* **116**, 76–86 (2017).

6. National Wildfire Coordinating Group, "NWCG Smoke Management Guide for Prescribed Fire" (2020).

7. A. Hsu, *et al.*, A global assemblage of regional prescribed burn records — GlobalRx. *Sci Data* **12**, 1083 (2025).

8. Z. Guo, *et al.*, Reconstructed global monthly burned area maps from 1901 to 2020. *Earth System Science Data* **17**, 3599–3618 (2025).

9. O. US EPA, 2020 National Emissions Inventory (NEI) Technical Support Document (TSD): Section 7 Fires – Wild, Prescribed, and Agricultural Field Burning. (2023). Available at: https://www.epa.gov/air-emissions-inventories/2020-national-emissions-inventory-nei-technical-support-document-tsd [Accessed 8 October 2025].

10. J. H. Scott, *et al.*, Wildfire Risk to Communities: Spatial datasets of landscape-wide wildfire risk components for the United States (2nd Edition). (2024).

11. K. E. Chung, *et al.*, Managing Smoke Risk from Wildland Fires: Northern California as a Case Study. *Environ. Sci. Technol.* **59**, 13912–13923 (2025).

12. F. L. M. Santos, *et al.*, Prescribed Burning Reduces Large, High-Intensity Wildfires and Emissions in the Brazilian Savanna. *Fire* **4**, 56 (2021).

13. K. Liao, J. Buch, K. D. Lamb, P. Gentine, Simulating the air quality impact of prescribed fires using graph neural network-based PM2.5 forecasts. *Environmental Data Science* **4**, e11 (2025).

14. J. Russell-Smith, *et al.*, Managing fire regimes in north Australian savannas: applying Aboriginal approaches to contemporary global problems. *Frontiers in Ecology and the Environment* **11**, e55–e63 (2013).

15. R. D. Ottmar, M. D. Schaaf, E. Alvarado, Smoke considerations for using fire in maintaining healthy forest ecosystems. *In: Hardy, Colin C.; Arno, Stephen F., eds. The use of fire in forest restoration. Gen. Tech. Rep. INT-GTR-341. Ogden, UT: U.S. Department of Agriculture, Forest Service, Intermountain Research Station. p. 24-25.* **341** (1996).

16. M. A. Storey, O. F. Price, Comparing the Effects of Wildfire and Hazard Reduction Burning Area on Air Quality in Sydney. *Atmosphere* **14**, 1657 (2023).

17. L. Volkova, S. H. Roxburgh, C. J. Weston, Effects of prescribed fire frequency on wildfire emissions and carbon sequestration in a fire adapted ecosystem using a comprehensive carbon model. *Journal of Environmental Management* **290**, 112673 (2021).

18. W. Guo, *et al.*, Enhancing carbon storage through proactively managing fire-prone coniferous mountain forests. *Ecological Modelling* **510**, 111332 (2025).

19. A. A. Ager, M. A. Finney, A. McMahan, J. Cathcart, Measuring the effect of fuel treatments on forest carbon using landscape risk analysis. *Natural Hazards and Earth System Sciences* **10**, 2515–2526 (2010).

20. L. A. Chiono, D. L. Fry, B. M. Collins, A. H. Chatfield, S. L. Stephens, Landscape-scale fuel treatment and wildfire impacts on carbon stocks and fire hazard in California spotted owl habitat. *Ecosphere. 8(1): e01648* **8**, e01648 (2017).

21. J. Hyde, E. K. Strand, J. Hyde, E. K. Strand, Comparing Modeled Emissions from Wildfire and Prescribed Burning of Post-Thinning Fuel: A Case Study of the 2016 Pioneer Fire. *Fire* **2** (2019).

22. J. W. Long, S. A. Drury, S. G. Evans, C. J. Maxwell, R. M. Scheller, Comparing smoke emissions and impacts under alternative forest management regimes. *Ecology and Society* **27** (2022).

23. C. Narayan, P. M. Fernandes, J. van Brusselen, A. Schuck, Potential for CO2 emissions mitigation in Europe through prescribed burning in the context of the Kyoto Protocol. *Forest Ecology and Management* **251**, 164–173 (2007).

24. M. Hurteau, M. North, Fuel treatment effects on tree-based forest carbon storage and emissions under modeled wildfire scenarios. *Frontiers in Ecology and the Environment 7: 409-414* **7**, 409–414 (2009).

25. C. Wiedinmyer, M. D. Hurteau, Prescribed fire as a means of reducing forest carbon emissions in the western United States. *Environ Sci Technol* **44**, 1926–1932 (2010).

26. T. Vilén, P. M. Fernandes, Forest Fires in Mediterranean Countries: CO2 Emissions and Mitigation Possibilities Through Prescribed Burning. *Environmental Management* **48**, 558–567 (2011).

27. G. E. Defossé, G. Loguercio, F. J. Oddi, J. C. Molina, P. D. Kraus, Potential CO2 emissions mitigation through forest prescribed burning: A case study in Patagonia, Argentina. *Forest Ecology and Management* **261**, 2243–2254 (2011).

28. L. Volkova, *et al.*, Fuel reduction burning mitigates wildfire effects on forest carbon and greenhouse gas emission. *Int. J. Wildland Fire* **23**, 771–780 (2014).

29. M. Burke, *et al.*, The changing risk and burden of wildfire in the United States. *Proceedings of the National Academy of Sciences* **118**, e2011048118 (2021).

30. M. Kelp, *et al.*, Prescribed Burns as a Tool to Mitigate Future Wildfire Smoke Exposure: Lessons for States and Rural Environmental Justice Communities. *Earth's Future* **11**, e2022EF003468 (2023).

31. L. Kiely, *et al.*, California Case Study of Wildfires and Prescribed Burns: PM2.5 Emissions, Concentrations, and Implications for Human Health. *Environ. Sci. Technol.* **58**, 5210–5219 (2024).

32. M. Kelp, *et al.*, Effect of Recent Prescribed Burning and Land Management on Wildfire Burn Severity and Smoke Emissions in the Western United States. *AGU Advances* **6**, e2025AV001682 (2025).

33. Z. Li, *et al.*, The Trade-offs between Wildfires and Prescribed Fires: A Case Study for 2016 Gatlinburg Wildfires. *ACS EST Air* **2**, 236–248 (2025).
